# Synergistic Effects of Warming and Internal Nutrient Loading Interfere with the Long-Term Stability of Lake Restoration and Induce Sudden Re-eutrophication

**DOI:** 10.1021/acs.est.2c07181

**Published:** 2023-02-20

**Authors:** Xiangzhen Kong, Maria Determann, Tobias Kuhlmann Andersen, Carolina Cerqueira Barbosa, Tallent Dadi, Annette B.G. Janssen, Ma. Cristina Paule-Mercado, Diego Guimarães Florencio Pujoni, Martin Schultze, Karsten Rinke

**Affiliations:** † State Key Laboratory of Lake Science and Environment, Nanjing Institute of Geography and Limnology, Chinese Academy of Sciences, 210008 Nanjing, China; ‡ Department of Lake Research, Helmholtz Centre for Environmental Research - UFZ, 39114 Magdeburg, Germany; § Department of Ecoscience, Aarhus University, 8000 Aarhus, Denmark; ∥ Zoology and Physiology Department, University of Wyoming, Laramie, Wyoming 82071, United States; ⊥ Water Systems and Global Change Group, Wageningen University & Research, Droevendaalsesteeg 3, 6708 PB, Wageningen, The Netherlands; # Institute of Hydrobiology, Biology Centre, Czech Academy of Sciences, Na Sádkách 7, České Budějovice 37005, Czech Republic; ¶ Laboratório de Limnologia, Ecotoxicologia e Ecologia Aquática, Instituto de Ciências Biológicas, Universidade Federal de Minas Gerais, Avenida Antônio Carlos 6627, Cep 31270-901 Belo Horizonte, Minas Gerais, Brazil

**Keywords:** eutrophication, cyanobacterial blooms, phosphorus precipitation, internal loading, climate change, GOTM-WET

## Abstract

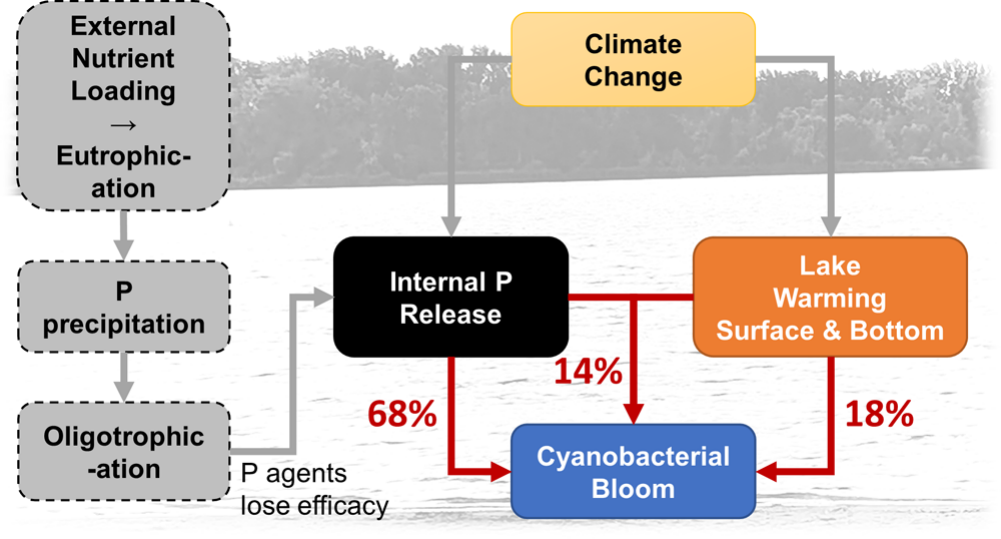

Phosphorus (P) precipitation is among the most effective treatments to mitigate lake eutrophication. However, after a period of high effectiveness, studies have shown possible re-eutrophication and the return of harmful algal blooms. While such abrupt ecological changes were attributed to the internal P loading, the role of lake warming and its potential synergistic effects with internal loading, thus far, has been understudied. Here, in a eutrophic lake in central Germany, we quantified the driving mechanisms of the abrupt re-eutrophication and cyanobacterial blooms in 2016 (30 years after the first P precipitation). A process-based lake ecosystem model (GOTM-WET) was established using a high-frequency monitoring data set covering contrasting trophic states. Model analyses suggested that the internal P release accounted for 68% of the cyanobacterial biomass proliferation, while lake warming contributed to 32%, including direct effects via promoting growth (18%) and synergistic effects via intensifying internal P loading (14%). The model further showed that the synergy was attributed to prolonged lake hypolimnion warming and oxygen depletion. Our study unravels the substantial role of lake warming in promoting cyanobacterial blooms in re-eutrophicated lakes. The warming effects on cyanobacteria via promoting internal loading need more attention in lake management, particularly for urban lakes.

## Abstract 2

Warming synergistically promotes re-eutrophication with internal nutrient loading and exacerbates cyanobacterial blooms in urban lakes 30 years after phosphorus mitigation.

## 1 Introduction

Freshwater lakes are of vast importance for human wellbeing, providing a wide span of ecological services such as recreation, irrigation, and drinking water supply.^1^ Small urban lakes are globally ubiquitous and are usually shallow and artificial.^2,3^ Many urban lakes are highly eutrophic^4^ associated with increasing frequency, duration, and magnitude of cyanobacterial blooms, posing widespread threats to ecological and human health.^5,6^ Excessive nutrient loading and climate warming have been acknowledged to promote cyanobacterial blooms.^7−9^ As such, tremendous investments in phosphorus (P) precipitation (e.g., using aluminum salts) are implemented to restore and sustain the urban lakes. Nevertheless, after the effective duration (spanning from a few months to 45 years), these lakes often suffer from re-eutrophication and abrupt cyanobacterial blooms.^10^ Thus far, knowledge remains limited to the causal effects of such re-eutrophication, and more importantly, of the consequential cyanobacterial blooms, in urban lakes.

Many urban lakes have low surface inflows and are fed by groundwater, particularly those gravel pit lakes in the urban area, pointing to rather low external loading from catchments, although diffusive P loading occurs if untreated wastewater inputs or other sources exist (e.g., from wild camping and bathing).^11^ It is therefore good management practice for such urban lakes to first minimize or exclude such uncontrolled nutrient inputs, in particular with respect to wastewater management, and only then apply P precipitation to reduce nutrient concentrations and trophic state. Although effective in many cases, these lakes may keep receiving low but continuous P inputs from the recreational activities over decades, resulting in high P accumulation in the sediments due to long water residence time and limited export. Therefore, these lakes bear the risk of rising internal P loading so that a reduction in external loading did not result in an everlasting recovery.^12^ Such activation of the internal loading, possibly occurring decades after the successful P precipitation,^13^ can be attributed to loss of the P adsorbent binding efficacy, loss of the macrophytes, and wind dredging^10,14^ and further stimulated by the low but constant P inputs from nontributary-driven external inputs like atmospheric deposition, birds, bathing, and groundwater inflow. In addition, the role of temperature on the internal nutrient flux has been understood at seasonal level^15^ but, to the best of our knowledge, has not been investigated at the multidecadal scale. To this end, this study aims to address the occurrence of such a sudden onset of internal P loading and evaluate its contribution to water quality deterioration. We hypothesize that such a “switch” from accumulating, low external loading into a sudden fortification of internal loading is the predominant trigger of lake re-eutrophication and cyanobacterial blooms after long times of successful restoration.^10^ In any case, such a switch in nutrient sources can occur at very short time scales and necessarily result in catastrophic changes in the lake ecosystem being highly relevant for lake managers.

Prevailing evidence of climate change impacts on the proliferation of cyanobacterial blooms^16,17^ points to the need of disentangling the contributions from climatic factors to the cyanobacterial blooms in lakes suffering from re-eutrophication. Recently, advances in climate modeling enable an elegant method of climate attribution by providing climate trajectories without the influence of anthropogenic activities on greenhouse gas dynamics as the so-called “piControl” scenario.^18^ Further advances in climate modeling allow us to project climate change at unprecedented spatial (<1° geodetic coordinates) and temporal resolution (daily), offering the possibility to integrate with recent developments of process-based lake ecosystem models.^19,20^ The attribution of lake ecosystem changes to climate change has been performed for several lake physical properties such as temperature and ice cover,^21^ yet the water quality proxies with considerable management concern (e.g., Chl-a, algal biomass) have not been well studied, especially in those re-eutrophicated lakes that underwent dramatic shifts at times where climatic factors also progressively changed.

The relative contribution of internal loading and lake warming, as well as their potential synergistic effects in modulating cyanobacterial bloom, remains largely unexplored. Lake warming may increase the water temperature in bottom layers and promote internal loading due to higher rates of mineralization and more frequent and/or prolonged anoxic conditions, leading to the synergistic effects of warming and internal loading on cyanobacterial blooms.^22,23^ Disentangling the role of lake warming and internal loading is challenging because cyanobacterial blooms are regulated by a complex mixture of external and internal controls.^24^ The prediction of cyanobacteria, therefore, requests a process-based ecological modeling approach incorporating key regulators of climate-driven physical dynamics and in-lake biogeochemical processes. Process-based ecosystem models provide the opportunity to bring the lake *in silico*, performing the “virtual lake” experiments that are usually costly and even infeasible in the field. Taken together, given the tremendous management concern for urban lakes, it is tempting to explore the rigorous attribution of warming and internal loading impact on the observed sudden shifts toward re-eutrophication and cyanobacterial blooms.

**Figure 1:**
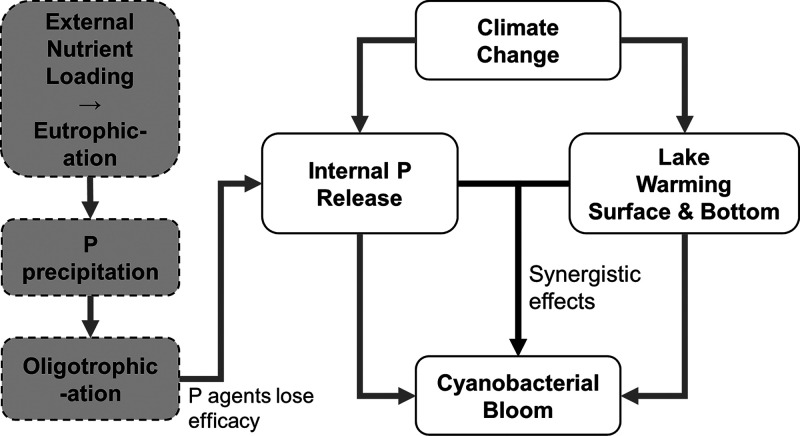
A conceptual diagram showing the hypotheses in the present study, i.e., the predicted synergistic effects of climate change and internal nutrient loading on cyanobacterial blooms in urban lakes fed by groundwater after the effective period of P precipitation (∼30 years) and rapid re-eutrophication.

In the present study, we aim to quantify the importance of lake warming and internal loading in driving the rapid re-eutrophication and cyanobacterial blooms in urban lakes with low external nutrient loading. We strategically selected a typical urban lake in central Germany (Lake Barleber),^25^ which was subject to a rapid re-eutrophication that occurred in 2016 after the large-scale P precipitation in 1986.^13^ Yet, the role of lake warming and its synergies with internal loading remains unknown. Our hypotheses are the following: (1) The lake re-eutrophication in 2016 was triggered by sudden internal P release due to both loss of binding agent efficacy and lake hypolimnetic warming. (2) The cyanobacterial blooms were driven by the synergistic effects of both increasing nutrients and lake warming (summarized in Figure 1). To address these hypotheses, we combine intensive field monitoring data with a process-based lake modeling approach. We deem that our results may provide new insights for urban lake eutrophication and mitigation under global change beyond the study site.

## 2 Materials and Methods

### 2.1 Study Site

**Figure 2:**
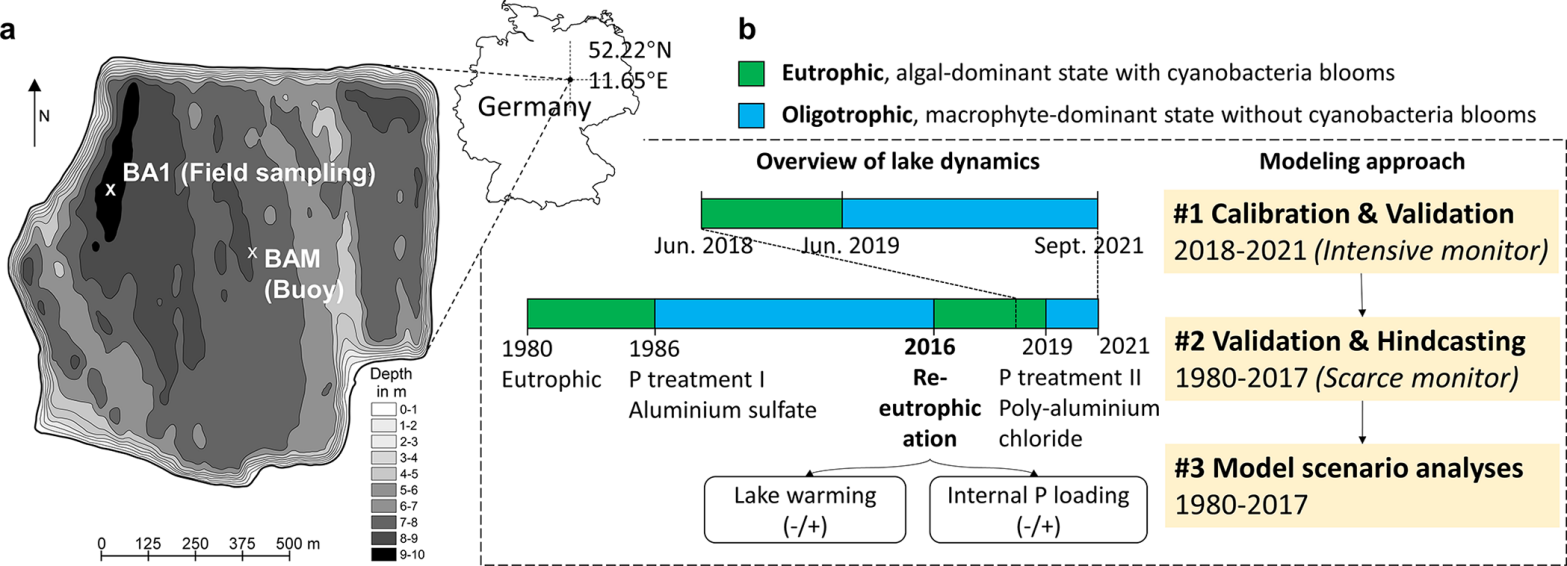
(a) Bathymetry map of Lake Barleber in central Germany with the location of the sampling site (BA1) in the deepest part of the lake and monitoring buoy site in the lake center (BAM). (b) A schematic diagram of the long-term ecosystem dynamics of Lake Barleber, together with an overview of the modeling approach in disentangling the role of lake warming and internal P loading in triggering the lake re-eutrophication in 2016.

Lake Barleber (52°13′15″N, 11°39′00″E) is located in northern Magdeburg city in central Germany (Figure 2). The lake was artificially created by gravel excavations in the 1930s, with a surface area of 103 ha, a maximum depth of 11 m, a mean depth of 6.7 m, and a water volume of ∼6.9 × 10^6^ m^3^.^26^ The lake is monomictic, i.e., stably stratified in summer and mixed in other seasons, except for intermittent ice cover in winter causing inverse stratification. The lake has no surface inflow and outflow but is receiving 640,000 m^3^·a^–1^ of groundwater inflows balanced by 530,000 m^3^·a^–1^ of groundwater outflow and ∼190 mm·a^–1^ more evaporation than precipitation, resulting in a water residence time of ca. 10 years.^26^ The lake’s surrounding area is dominated by recreational use (camping ground, weekend cottages, beaches) while the wider catchment of the groundwater entering Lake Barleber is dominated by the cultivation of forest, grains, and root vegetables (Table S1).^13^ During the past decades, the lake shifted between a clear oligotrophic and a turbid eutrophic state. Soluble reactive phosphorus (SRP) concentrations remained low during the 1950s (clear water with high macrophytes coverage), gradually increased to ∼50 μg·L^–1^ during the 1970s, and rapidly enhanced to 150 μg·L^–1^ during the 1980s, causing cyanobacterial blooms. A lake-wide P precipitation using aluminum sulfate additions was performed in 1986, which switched the lake back to a clear-water state.^13^ However, the lake became eutrophic again in 2016 with the occurrence of summer cyanobacterial blooms; thus, another lake-wide P precipitation using poly(aluminum chloride) was implemented in July 2019, which again restored the lake to a clear-water state.

### 2.2 Data Collection

Meteorological data were collected from the ERA5 global meteorological reanalysis data (with a half-degree spatial resolution, provided by European Centre for Medium-Range Weather Forecasts, ECMWF) from 1979 to 2021 on an hourly basis from January 1979 to December 2021, including both a calibration and a validation period (2018 to 2021) and the long-term simulation period (1979 to 2017). Variables included wind speed at both E-W and N-S directions (m·s^–1^), air temperature (°C), air pressure (hPa), relative humidity (%), dew-point temperature (°C), solar radiation (W·m^–2^), and precipitation (mm·h^–1^). Cloud cover was calculated by latitude, air temperature, relative humidity, and short-wave radiation using the “calc_cc” function in the “gotmtool” package.^27^ We further collected meteorological data from 1979 to 2021 via the German meteorology service (DWD) at the station “Magdeburg” (ca. 15 km south of the lake) using the R package “rdwd”^28^ and meteorological data starting in June 2018 from a weather station on the monitoring buoy (BAM, Figure 2) reflecting the actual conditions on the lake. Both DWD and buoy data cannot exclusively be used for lake modeling due to long gaps, and for reasons of consistency, we favored the usage of ERA5. The ERA5 data were first corrected against the DWD data available from 1979 to 2021. Then, for a subset of meteorological variables from the weather station data on the lake buoy available from 2018 to 2021, the DWD data were corrected against the weather station data and further applied to the ERA5 data. The bias correction was performed using the linear regression model (Figures S1 and S2, Table S2).

From June 2018 to September 2021, water samples were taken at five depths (0.5, 2.5, 5.0, 7.0, and 9.0 m) from the deepest point in the lake (BA1) at biweekly intervals. Wet chemical analysis was performed in the laboratory using standard methods to determine total nitrogen (TN), nitrate nitrogen (NO_3_-N), ammonium nitrogen (NH_4_-N), total phosphorus (TP), dissolved P (DP), SRP, and dissolved silicon (Si) concentrations. For details on the methods, we refer to refs (25 and 29). In addition, we measured vertical profiles (at BA1) of water temperature, dissolved oxygen with a multiparameter probe (CTD90, Seas & Sun Technologies, Germany), and chlorophyll-a fluorescence with phytoplankton composition details of diatoms, green algae, and cyanophytes^30^ with a multichannel fluorescence probe (Fluoroprobe, BBE Moldaenke, Germany), at a biweekly basis with approximately 0.1 m vertical resolution. All probe data were linearly interpolated at 0.1 m intervals from the lake surface to the bottom. In addition, surface sediment chemical data including different forms of N and P during the summer of 2017 and early 2018 were collected from ref (31).

### 2.3 Lake Ecosystem Model Configuration

We used the coupled 1-dimensional General Ocean Turbulence Model (GOTM)^32^ and Water Ecosystem Tool (WET)^33^ to predict the lake ecosystem dynamics. GOTM was applied to simulate the hydrodynamic processes and thermal structure along the vertical gradient of the water column. The lake branch of GOTM is used, which differs from the default configuration by including the lake hypsography to specify depth-area relations.^34^ WET is based on the widely used 0-dimensional shallow lake ecosystem model PCLake^35^ and FABM-PCLake^36^ with a fully closed biogeochemical cycling for nutrients (nitrogen, phosphorus, and silicate). We configured WET with a typical food web of temperate lakes with three phytoplankton groups (diatoms, green algae, and cyanobacteria, in both water and sediment, including features such as buoyancy of cyanobacteria) and three additional trophic levels (zooplankton, zoobenthos, and fish) that constitute the dominant food web components. GOTM is coupled to WET by the Framework of Aquatic Biogeochemical Models (FABM)^37^ allowing feedback between both models.

Due to low fluctuations in water levels (41.33 ± 0.31 m a.s.l. from 1979 to 2021), the lake was modeled with constant depth (11 m). Groundwater was the only inflow estimated at a constant rate of 0.02 m^3^·s^–1^ (640,000 m^3^·a^–1^), which enters the bottom water layer in the model. In 2017 (eutrophic state), the annual TP loading from the catchment was estimated as 78 kg P·a^–1^ including 42 kg P·a^–1^ from atmospheric deposition (particulate), 26 kg P·a^–1^ from groundwater input, and 10 kg P·a^–1^ from diffusive input (recreation activities, e.g., bathing).^26^ In addition, internal P loading from the sediment amounted to 600 kg P·a^–1^ sustaining the high P level in the lake.^31^ These data were used to determine the parameters of inflow P concentrations and sediment P release rate (7.2 × 10^–5^ m^2^ d^–1^) in the model. To simulate the P-capping of the sediment by the P precipitation in July 2019, the parameter of the P diffusion rate was reduced by 3 orders of magnitude and kept low afterward (manually calibrated using the “restart” function in GOTM; SI text), and the state variables of SRP, dissolved organic P, and particulate P in the water phase were all reduced to the observed level (using the “manipulation” function in GOTM; SI text). Note that the modeled P diffusion flux varies over time, modulated by not only the diffusion rate constant but also other factors such as water temperature and dissolved oxygen (SI text). Besides, we used the export coefficient model (SI text) as a watershed nutrient export model to estimate the annual TN^38,39^ (Table S1) and dissolved silica^39^ loading to the lake from 1980 to 2021, considering a mixture of agricultural and natural vegetation as the main land use type in the drainage area.^13^ The export coefficient model is manageable and relatively simple, allowing it to be widely used to predict nutrient loss from catchments.^40^ An additional TN input from atmospheric deposition (at a rate of 18.1 kg·ha^–1^·a^–1^)^41^ was added to the TN loading to the lake.

### 2.4 Model Calibration and Validation

Before model calibration, initial values of the nutrient concentrations (different forms of N, P, and Si) in water and sediment surface were determined based on the field data in 2018^13,25^ to represent the sediment nutrient pool accurately. The model was calibrated and validated against the monitoring data set (+3 years from June 2018– September 2021) preceded by a 10-year spin-up period (2008–2018) equivalent to the lake water residence time. Monitoring data from June 2018 to June 2019 (before the P precipitation) were used for calibration, and data after the P precipitation were used for validation. We consider it as a strength to (1) test the process-based lake model against the case with actual restoration measures^42^ and (2) calibrate and validate the model with respect to a large intervention such as P precipitation, because a good performance in both stages would build trust in the model regarding a broad application domain under distinct lake ecological states. The Python-based program “PARSAC”^43^ was utilized to perform an automatic parameter optimization following a “bottom-up” approach (SI text and Table S3), which has been applied successfully in several applications.^44−46^ The parameters are reported in Table S4.

Model performance was evaluated based on the correlation coefficient (*R*) and relative error (*RE*).^47^ We determined the model performances for different state variables and classified them into four categories (fair, satisfactory, good, and excellent). This method was established by the evaluation of 100+ aquatic ecosystem model studies (from 1990 to 2002) based on *R* and *RE*(48) and was based on three preselected percentile thresholds arising from the 100+ models’ performances (20%, 50%, and 80% percentiles; see Table S5 for details). For example, for water temperature, we consider the model performance “satisfactory” if the *R* and *RE* values are in the range between 20 and 50% of the 100+ models’ performances. If *R* and *RE* provide different categories, median or lower level will be adopted (e.g., satisfactory (*R*) + good (*RE*) = satisfactory or satisfactory (*R*) + excellent (*RE*) = good).

### 2.5 Hindcast and Scenario Analyses from 1980 to 2018

We further extend the model simulation duration from 1980 to 2017 for hindcast. Additional water quality data were collected from ref (13) for further validation. These data include annual average TP, SRP, Chl-a concentration, and cyanobacterial biomass across the water column (0–7.5m) from 1983 to 2016 and zooplankton biomass for *Daphnia* and meso-zooplankton groups from 1986 to 1992. Both the P precipitation in autumn 1986 and the re-eutrophication in 2016 were modeled by manipulating the P diffusion rate, the same strategy as that for summer 2019 (Section 2.3) with the “restart” and “manipulation” functions (SI text). For water inflow, the discharge rate was kept constant at 0.02 m^3^·s^–1^.

In addition, we designed a scenario analysis from 1980 to 2021 to disentangle the effect of lake warming and internal P loading on the re-eutrophication in 2016. The ERA5 historical data used above was considered as the factual climate condition reflecting the warming, termed as clim+. For climate conditions without anthropogenic climate change (clim−), data of the “piControl” scenario from the Inter-Sectoral Impact Model Intercomparison Project (ISI-MIP)^18^ were collected based on four Global Climate Models (GCMs) (HadGEM2-ES, IPSL-CM5A-LR, MIROC-ESM-CHEM, GFDL-ESM2M) (Figure S3). In addition, internal P loading was set either as the same as before 2019 to reflect the occurrence of internal loading (intP+) or without internal loading as mentioned in Section 2.3 (intP−). The two-way ANOVA test was used to determine the relative contribution of climate change (clim −/+) and internal loading (intP −/+) to the cyanobacterial biomass in the surface water (0–1 m depth) from 2016 to 2018. The Mann–Kendall test and Sen’s slope were utilized to determine the temporal trends and rates of changes, respectively, using the R package “trend”.^49^ All statistical analyses were performed in R version 4.0.5.^50^

## 3 Results

### 3.1 Model Calibration and Validation (2018–2021)

**Figure 3:**
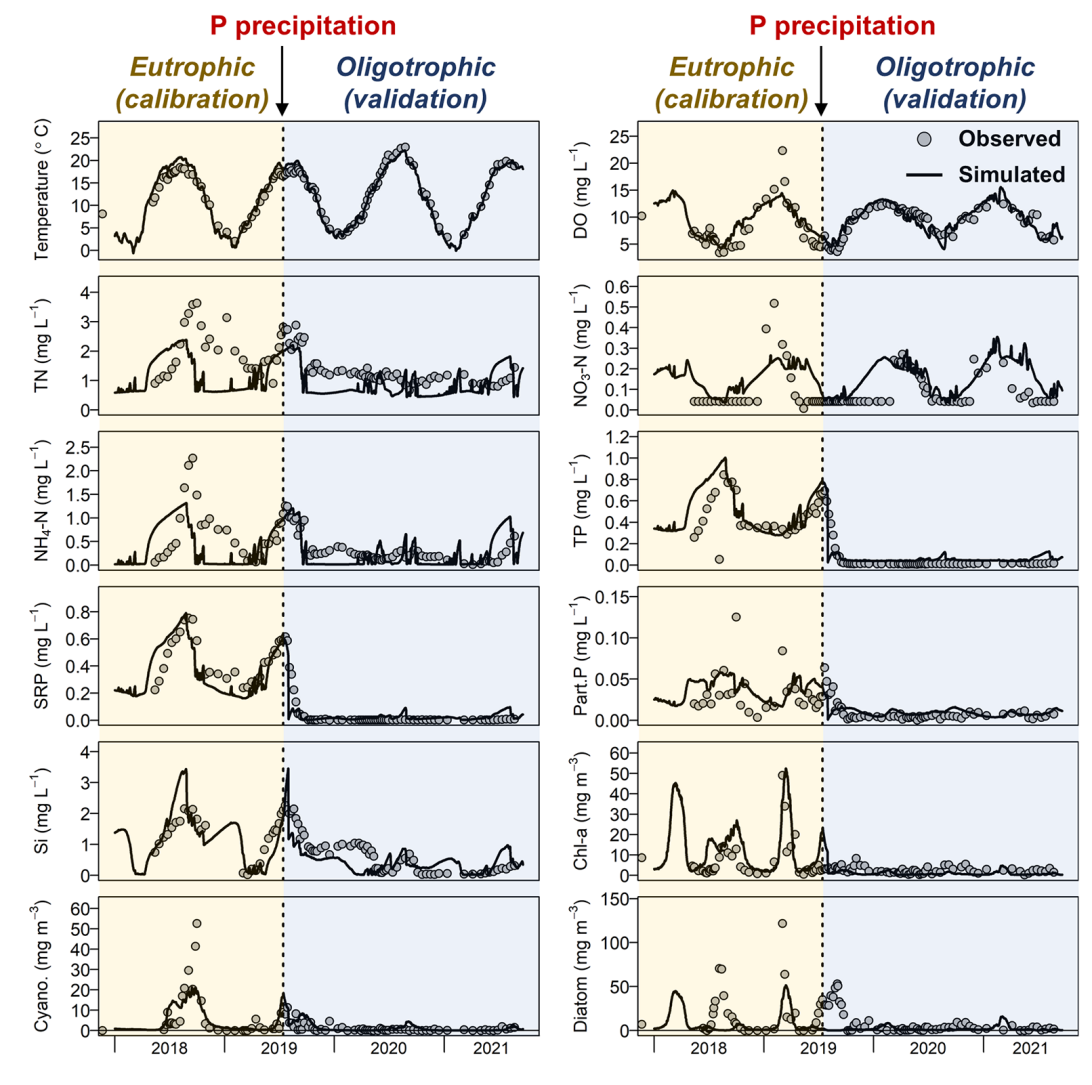
Volume-weighted values of observed and simulated water quality data across the whole water column (0–10 m depth) from 2018 to 2021 in Lake Barleber. The model was calibrated against the data between June 2018 and June 2019 when the lake was eutrophic (before the P precipitation) and validated against the data between July 2019 and November 2021 when the lake was oligotrophic. Note that the abundance of cyanobacteria and diatom are shown by Chl-a fraction of the total biomass (in the units of mg·m^–3^).

Our model showed “good” performance for most state variables during the calibration and validation periods in Lake Barleber (Figure 3, Table S6), indicating adequate predictive power of the model for the lake ecosystem dynamics under contrasting states (eutro-/oligotrophic).

For water temperature, both magnitudes and seasonal patterns across the water column were well-reproduced (Figure S4), with “good” and “excellent” performance for calibration and validation (Table S6). The model accurately reflected increased water transparency and heat penetration via deeper reaching short wave radiation to the hypolimnion after the P precipitation. Therefore, the bottom temperature in the summer of 2020 was significantly higher than in the preceding years.

Modeled performance for DO was “good” for both calibration and validation. The hypoxia was reduced after the P precipitation, resulting in a higher summer mean DO concentration in the water column after 2019 (Figure 3). Only the single DO measurement peaking in winter 2019 during the diatom bloom was not fully captured.

Nitrogen (including TN, NO_3_-N, and NH_4_-N) was modeled with a “good” performance in calibration and validation periods except for NO_3_-N (fair). The model marginally overestimated the NO_3_-N concentration but reproduced the timing and magnitude of the peaks in winter. NH_4_-N was well modeled attributing to the accurate prediction of sediment release (Figure S5). However, the model slightly underestimated the NH_4_-N concentration in the epilimnion in the first year (Figure S6).

The magnitude and variations in TP, SRP, dissolved organic P, and particulate P were all modeled with “good” performance and “excellent” for TP during the validation period (Figure 3, Table S6). Before P precipitation, TP concentration was high in the hypolimnion during summer (up to 1.5 mg·L^–1^) due to substantial sediment release that sustained the excessive P in the pelagic environment (Figure S5). The model nicely mimicked the P mitigation in the water column after P precipitation. In addition, Si concentration was modeled with “good” performance. The overarching pattern of low Si levels in winter and high in summer was well-captured (Figure 3).

Chl-a concentration, as well as cyanobacterial and diatom biomass, were reasonably modeled mostly with “good” performance (only the diatom calibration was “satisfactory”). The model successfully captured the reduction in Chl-a and algal abundance after the P precipitation. Specifically, the model captured the summer peaks of cyanobacteria and winter peaks of the diatoms. The diatom signals in the summer 2018 and 2019 were not captured by the model likely because these signals overlap with other algal species (e.g., mixotrophic dinoflagellate) that cannot be distinguished.^25^ The model nevertheless correctly showed that diatom and cyanobacteria were the dominant phytoplankton groups before the P precipitation, accounting for the majority of Chl-a.

### 3.2 Long-Term Simulation of Lake Ecosystem Dynamics (1980–2021)

**Figure 4:**
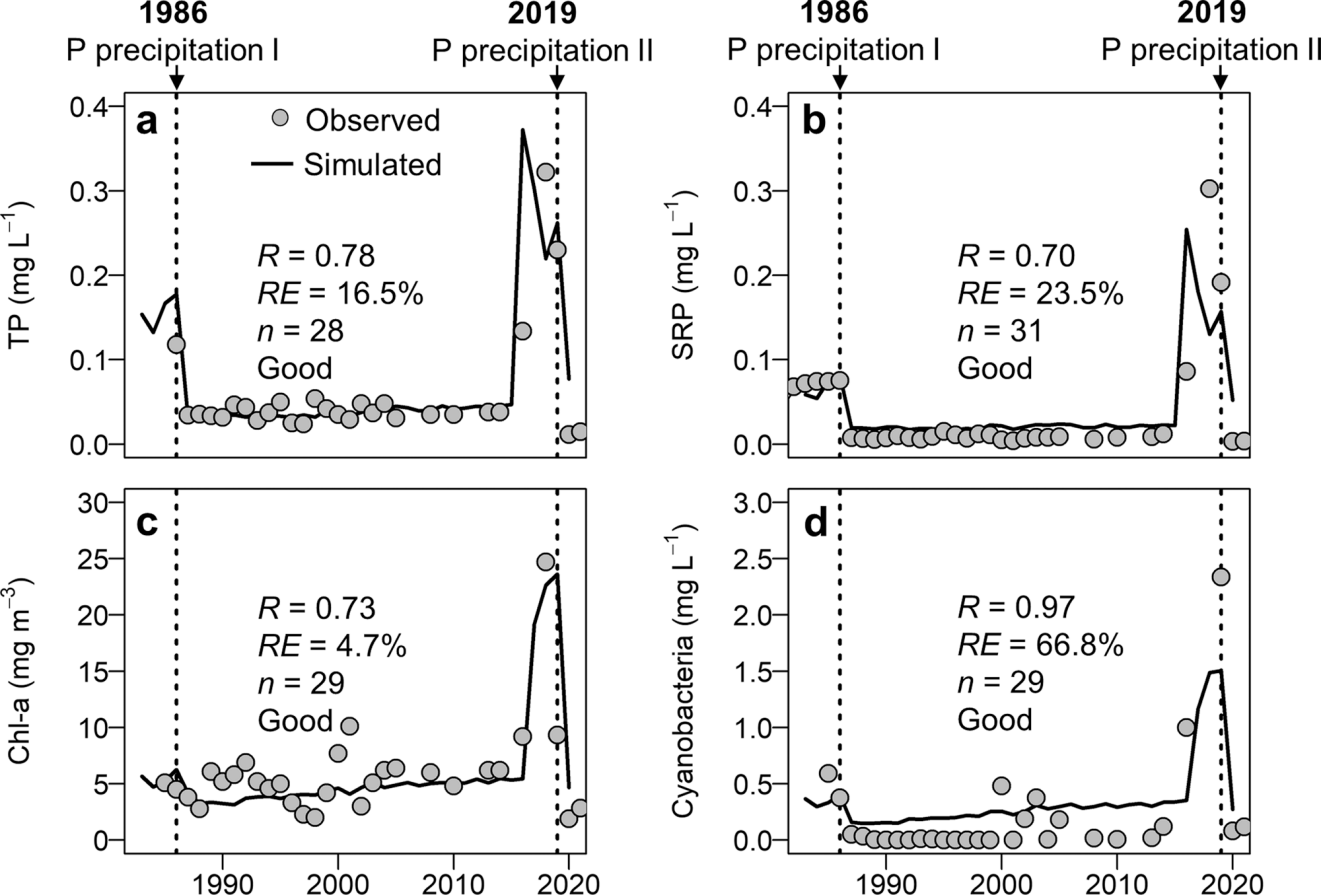
Comparison between observed and simulated annually average nutrient concentrations and phytoplankton abundance in the water column from 1982 to 2021 in Lake Barleber. (a) TP concentration. (b) Soluble reactive phosphorus (SRP) concentrations. (c) Chl-a concentration. (d) Cyanobacterial biomass. Volume-weighted values of observed and simulated data across the water column (0–7.5 m) are provided. Vertical dashed lines represent the two P precipitations. Data collected from ref (13).

The outcomes of the long-term simulation from 1980 to 2021 fitted well with the observations of nutrients (P) and algae (Chl-a concentration and cyanobacterial biomass), all with “good” performance based on *R* and *RE* (Figure 4). Driven by internal P loading variations, the model accurately captured the timing and, more importantly, the magnitude of shifts in P concentration and algae biomass in the water column after P precipitations (both 1986 and 2019) and re-eutrophication (2016). In addition, our model accurately predicted the dominance of *Daphnia* and the minor role of meso-zooplankton (including ciliates and rotifers) from 1986 to 1992 (Figure S7). Zoobenthos and fish dynamics are not verified due to a lack of data, and future evaluation is required. The “good” performance of the long-term model simulation serves as an additional validation, demonstrating the model’s capacity to simulate the lake ecosystem dynamics over decades including major engineering efforts and their consequences on the ecosystem level.

Further, our model predicted the long-term dynamics of lake water temperature, dissolved oxygen, and concentrations of various nitrogen species (Figures S8 and S9). From 1980 to 2021, simulated epilimnion and hypolimnion water temperatures increased significantly by 0.048 and 0.007 °C·a^–1^, respectively (Mann–Kendall test, *p* < 0.005). The whole lake was warming at a rate of 0.019 °C·a^–1^ (Mann–Kendall test, *p* < 0.001). For DO, simulated results pointed to a decreasing trend since 1980 (Figure S8) with rates of −0.011 and −0.023 mg·L^–1^·a^–1^ in the epi- and hypolimnion, respectively, and −0.021 mg·L^–1^·a^–1^ for the whole lake (Mann–Kendall test, *p* < 0.001). For N concentrations, simulated TN, NO_3_-N, and NH_4_-N all increased after the P precipitation in 1986 (Mann–Kendall test, *p* < 0.001) and the second P precipitation in 2019 (Figure S9). These results imply the interactions between long-term biogeochemical cycling of both N and P as decreasing algal growth due to P reduction also reduces N-uptake by algae, leaving a higher fraction of N in the inorganic fraction.

### 3.3 Scenario Analyses on Key Drivers of Re-eutrophication

**Figure 5:**
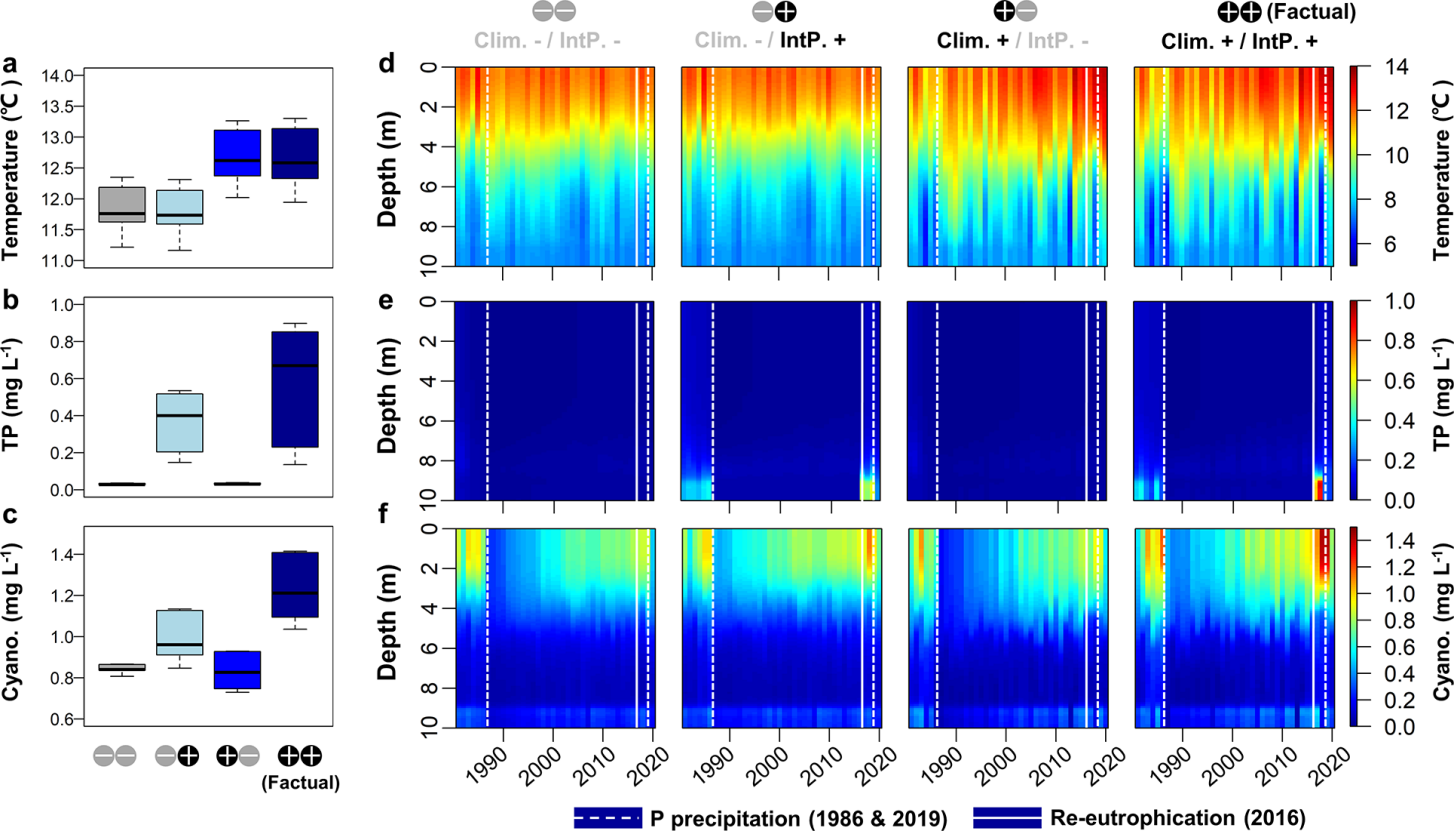
Scenario analyses of the long-term ecosystem dynamics in Lake Barleber. (a–c, from top to bottom) Lake water temperature (0–2 m in depth), water TP concentration (8–10 m in depth), and cyanobacterial biomass (0–1 m in depth) from 2016 to 2018, i.e., the re-eutrophication stage before the second P precipitation in 2019. (d–f, from top to bottom) Vertical profiles of lake water temperature, TP concentration,and cyanobacterial biomass (1981–2020) under different scenarios. Climate change (clim. −/+) and internal P release (intp. −/+) were the two factors explored, resulting in a two-factorial experimental design. Note that for the “clim–” condition, projections from four climate models were used and the average lake model outputs are shown here. “Factual” represents the actual and historical situation that occurred in the lake, with both climate change and internal P loading.

Scenario analysis revealed that the internal P release and lake warming synergistically reinforced cyanobacterial blooming from 2016 to 2018 (Figure 5). The annual average of cyanobacterial biomass in surface water was increased by 46.1% compared to the scenario without lake warming and internal P loading. For such an increase in cyanobacteria, the model analyses showed that internal P release accounted for 68%, lake warming contributed to 18%, and the interactive impacts of both lake warming and internal P release accounted for the remaining 14%. The model quantifies the processes driving the re-eutrophication and cyanobacterial bloom in three aspects: First, the sudden boost of internal P loading in 2016 resulted in a rapid increase of TP concentration in the hypolimnion (from 0.03 to 0.56 mg·L^–1^) after being stabilized for over 30 years (Figure 5). Such an increase of bottom P concentration further led to increasing epilimnion P concentration during the recirculation period in winter 2016, thereby promoting the cyanobacterial blooms in the consecutive summers of 2017 and 2018 (Figure 5). Second, lake warming further promoted the cyanobacterial bloom and exaggerated the water quality deterioration. Water temperature in the epilimnion was on average 0.9 °C higher than the condition without the warming effect (Figure 5), which accumulated over time as a gradual contributing factor (Figure S8). Third, synergistic interaction between lake warming and internal P loading was apparent because the promotion of the cyanobacterial blooms by lake warming (clim+) was only significant under sediment P release (intP+, but not intP−) (Figure 5). Lake warming enhanced the magnitude of sediment P release in 2016, resulting in 53.6% higher TP concentration and 23.5% more cyanobacterial biomass.

## 4 Discussion

### 4.1 Synergistic Effects of Lake Warming and Internal Loading in Re-eutrophication

Despite the well-acknowledged importance of lake warming on cyanobacterial blooms,^7^ such impact remains largely overlooked as a driver of re-eutrophication and cyanobacterial blooms in many urban lakes. This neglection was mainly due to the difficulty in quantifying the contribution of warming to the cyanobacterial blooms, which was somehow “masked” by the predominant influence of the internal loading. Here, we sought to fill in the gap by using a process-based modeling approach in a lake experiencing sudden shifts in trophic states by re-eutrophication at a time scale of 30 years. We confirmed the hypotheses that the re-eutrophication and cyanobacterial blooms from 2016 to 2019 were driven by the synergistic effects of both increasing internal nutrients loading and lake warming and estimated that lake warming has contributed to 32% of the cyanobacterial biomass. Thus, we anticipate that, in the context of global warming, lake water quality would worsen due to more severe cyanobacterial blooms after re-eutrophication.

One advantage of the modeling approach is to disentangle a complex mixture of direct and indirect causal-effect pathways in driving the cyanobacterial bloom based on comprehensive knowledge of the phenomenon.^7^

First, the model showed warmer surface water under factual climate (with warming) than the condition driven by preindustrial climate, which increased the growth rate of cyanobacteria and promoted their advantage over other species under higher temperature.^24^ Nevertheless, the growth promotion due to temperature increase may not be the sole factor because the scale of warming (+0.9 °C) would enhance the Arrhenius temperature modifier on the growth rate by ∼10%. Thus, temperature increase is a confounding factor driving cyanobacteria blooms. The higher water temperature enhances water stratification strength,^9^ and such increased stabilization of the water column favored cyanobacteria due to their physical trait of being buoyant. In addition, more intensive biogeochemical processes in the water column due to warming (e.g., higher nutrient availability due to more rapid mineralization) would increase the cyanobacteria biomass. These mechanisms in promoting cyanobacteria growth have been incorporated by the GOTM-WET^33^ and jointly contributed to 18% of the cyanobacterial biomass.

Second, our model unravels the synergistic effect between climate warming and internal loading, which accounted for 14% of the cyanobacterial biomass. Based on existing evidence,^54,55^ we assume that the synergy is predominantly induced by the enhanced internal loading due to warmer hypolimnion (i.e., higher diffusion from sediment and water) and/or stronger hypoxia-driven P release from the sediment. Our model indicated a significantly warming hypolimnion in the lake from 1980 to 2021 with a rate of 0.07 °C·decade^–1^, which was very close to the median level among lakes around the globe (0.06 °C·decade^–1^).^56^ Such warming in lake hypolimnion was in parallel to a decreasing oxygen concentration based on model simulations (Figure S8). Therefore, it is likely that the interaction between lake warming and internal loading arises from enhanced sediment P loading due to both warmer hypolimnion and stronger oxygen depletion. We zoom in on the re-eutrophication event in the summer 2017 and analyze the simulated internal P flux together with water temperature and DO concentration under both “clim–” and “clim+” scenarios (Figure S10). The model results suggest that, compared to the condition without climate warming (“clim-”), the factual warming climate (“clim+”) leads to higher internal P flux from −0.59–3.04 to 1.23–12.92 mg·m^–2^·d^–1^, jointly with a prolonged period of higher water temperature and a persistent DO depletion in the hypolimnion during summer (Figure S10a–c). Given that the internal P flux is modeled with an Arrhenius temperature modifier and oxygen dependence (SI text), we could confirm these mechanisms potentially driving the synergistic effects from the process-based modeling. The increasing extent of lake hypoxia has been reported globally,^23,57^ and our results further suggest that it may promote internal nutrient loading and thereby cyanobacterial blooms. In addition, a recent study proposes that the cyanobacterial bloom may formulate a positive feedback loop by increasing sediment pH and reducing bottom DO, to enhance internal nutrient loading and sustain the bloom, which could be further enhanced by climate extremes such as heatwave or precipitations.^58^ This process may also contribute to the prolonged hypoxia in Lake Barleber because the duration of the hypoxia is predicted to be stronger in 2017 with a higher extent of cyanobacterial bloom (Figure S10d). Notably, the model predictions suggest that lake warming not only increases the peak biomass of cyanobacteria but also prolongs the duration of the cyanobacterial bloom (Figure S10d), particularly in summer 2017, which could be the result of the stronger internal loading fluctuation.^22^

Our findings would be relevant for many other lakes. First, the small, gravel pit lakes in urban area like Lake Barleber are ubiquitous around the globe due to high demand of gravel and sand for construction (1.7 × 10^8^ metric tons·a^–1^).^4,51,52^ Second, many of these lakes suffer from severe eutrophication and water quality deterioration. For nearly half a century, P precipitation by aluminum agents has been used to reduce P levels in these lakes as an effective measure for ecological restoration.^10,53^ Third, our previous study in the same lake shows that the process-based lake model is directly applicable to another natural, urban lake (Lake Müggelsee fed by the Spree River in Germany). Such model transfer without any further modifications suggests that the present modeling work would be transferable to other types of urban lakes because our model can capture general processes and patterns. Lastly, our findings regarding the quantification of contribution from climate warming and internal P loading (and their synergy) are valuable for decision making and climate adaptation, particularly the need for more efforts in lake restoration in the context of the unprecedented global warming and the increasing occurrence of devastating re-eutrophication. The method integrating high-frequency monitoring and process-based ecosystem modeling is a powerful tool for future lake research.

### 4.2 The New Model Evaluation Method

We proposed a new evaluation method for the aquatic ecosystem model performance, which may help modeling studies to adequately describe how their models perform (Tables S5 and S6). Previous modeling approaches usually evaluate the performance based on certain criteria such as *RMSE*, *RE*, or coefficient of determination.^47^ However, it is often difficult to determine if the performance is acceptable and to justify how well the model works for the system. Based on the statistical analyses of existing model performance criteria,^48^ we established a new, simple method to offer performance metrics on each model variable with different levels. This method would be particularly helpful to (1) determine when the model calibration is adequate to stop and (2) communicate the modeling performance and facilitate the comparison of the lake model studies.

However, there are several limitations to the current method. First, the criteria thresholds are based on the studies before 2004^48^ so many recent model studies are not included. Second, the percentiles to determine different performance levels (Fair (<20%), Satisfactory (20–50%), Good (50–80%), and Excellent (80–100%); see Table S5) are arbitrarily selected so that additional evaluation is needed. The adequacy of a certain model also relies on the purpose of modeling, which is not fully captured by this evaluation framework. Third, notably, the evaluation results are better with *RE* than *R* (Table S6). This suggests that the model could well-capture the magnitude of the variables (by *RE*) but is less accurate in temporal dynamics (by *R*). Thus, it remains for further discussion on how to decide the model performance when different performance metrics provide different criteria (e.g., “satisfactory” by *R* and “excellent” by *RE*). Finally, the scope (variable number), duration, frequency, and corresponding ecological states of the field data are not considered. A weighting factor would be ideal, such that field data with more variables (e.g., different nitrogen species rather than TN alone), longer duration, and higher frequency have higher weights because they provide more information for the model, and field data collected during the engineered, oligotrophic phase (e.g., after P precipitation) should be assigned with lower weights because the value could be close to detection limits and bring more uncertainty to the model.^59^

### 4.3 Strengths and Limitations of the Modeling

The strengths of the present modeling approach are reflected in both the short-term parametrization phase and the long-term hindcasting phase. First, the intensive field monitoring data before (2018–2019) and after (2019–2021) the P precipitation allows the GOTM-WET to be calibrated and validated against distinct ecosystem states. This provides a unique opportunity to enhance the robustness of the model toward both eutrophic and oligotrophic states. In addition, the model is capable of reflecting ecosystem-wide consequences of the P precipitation, which is an important step forward for the lake modeling application in evaluating lake restoration efficiency. The model’s capacity to simulate not only P but also N cycling (nitrate and ammonia) further points to a high model skill in reflecting the complex biogeochemical transformation highly relevant for re-eutrophication and cyanobacterial modeling (e.g., mineralization, denitrification, redox reactions, nutrient uptake). This promotes the model reliability during the long-term simulation from 1980 to 2021 driven by different climate forcings and varied magnitudes of external nutrient inputs. Second, our lake modeling approach for water quality embraces the long-term perspective over multiple decades (40+ years), which is among the minority cases at such a time scale^45,46,60^ and provides the opportunity to understand the lake ecosystem dynamics and their drivers at a much larger time scale.^20^ The model simulation results not only showed reasonable consistency with the field data (as an additional validation; Figure 4) but also constituted a quantitative data set of the lake ecosystem dynamics to explore, including both biogeochemical and ecological components (Figures S8 and S9 as examples). Furthermore, scenario analyses over the long-term historical period serve as the “virtual lake” to provide guiding hypotheses on the main drivers of the ecosystem dynamics.^46^ Taken together, we exemplify the modeling parametrization strategy when historical observations are scarce and recent monitoring is intensive and demonstrate the value of numerical ecological modeling in promoting our understanding of lake ecosystem changes under global changes.

Our modeling approach has limitations regarding the description of groundwater exchange and P precipitation. First, groundwater was considered as inflow into the lowest layer of the lake model, whereas it may enter the top layer and feed directly into the euphotic zone. The flow was considered constant based on the data from a dry period during 2018/2019, while seasonal and interannual variations have not been incorporated yet. Second, we modeled the P removal and release before and after the Al treatment in both 1986 and 2019 by manipulating the state variables and parameters in the model. For the parametrization of P release from the sediment, we calibrated the parameters based on the *in situ* measurements of internal P flux. In fact, this has been advocated for environmental modeling advances, that is, to parametrizing models to match measurements of not only for state variables (e.g., P concentration) but also for processes (e.g., internal P flux).^59^ Thus far, model studies on simulating the synergistic effect of the warming and internal P loads on cyanobacteria blooms after lake re-eutrophication are scarce, and our study is among the few to explicitly address this gap.^53,61^ Nevertheless, the P binding agent (Al) and the related factors (e.g., pH)^53,61^ have not been effectively modeled at a process level so that lake hydrodynamics and P binding to Al are not directly linked, which is warranted to be incorporated into WET in the future. Also, the effects of macrophytes on the internal P release were not fully evaluated due to a lack of historical records,^13^ which could be important given the significant differences in P cycling between algae- and macrophytes-dominant lakes.^62^ After P precipitation, recovery of macrophytes may stabilize the sediment, enhance the P storage in the plants/sediments as an additional buffer of P, and reduce the P release to the water column. These limitations make it difficult to estimate the effective duration of the P precipitation, which is, however, relevant for lake management. Overall, though these aspects cannot be considered for now due to data paucity, future works would be highly valuable to refine the groundwater and P-binding agent for improving the precision and function of the model.

### 4.4 Implications

Our modeling approach highlights the importance of the synergistic effects of both external (climate change) and internal forcing (sediment nutrient loading) in driving re-eutrophication in urban lakes. We verified such synergy in an urban lake in Germany 30 years after the P precipitation. These findings imply that the risk of re-eutrophication and the extent of cyanobacterial blooms are expected to be exacerbated by warming. This has far-reaching consequences for lake restoration and management as the nutrient targets we applied so far to reach or maintain a certain trophic state will not work in a far warmer future and need to be adjusted, i.e., stronger nutrient level reduction and higher efforts in restoration are demanded. Nutrient mitigation via chemical agents can help but is effective only over a certain period. Further, our modeling approach exemplifies how process-based modeling can quantify the contribution of various influential factors with complex interactive mechanisms. Improvements in the model are needed to substantiate the capacity in simulating the chemical agents (e.g., Al) to reinforce urban lake restoration and management.

## Data Availability

The field data supporting this paper are available from the Zenodo online repository: 10.5281/zenodo.7580961.

## Supporting Information Available

Additional details of the modeling approach, annual TN loading estimation for Lake Barleber, model coefficients, work steps for GOTM-WET calibration, threshold values of the correlation coefficient and relative error for lake ecosystem model performance for various variables, model evaluation, meteorology data and projections, observed and simulated water temperature profiles, volume-weighted values of observed and simulated water quality data, comparison between observed and simulated biomass of daphnia and meso-zooplankton, simulated water temperature and DO, TN, NO_3_-N, and NH_4_-N concentrations in the water column, and scenario analyses from 2015 to 2017 before and after the 2016 re-eutrophication of Lake Barleber (PDF)
Parameter explanation and the calibrated values of the GOTM-WET model from this study for Lake Barleber compared with the values from literature (XLSX)
