# Supplementary material for: Synergistic Effects of Warming and Internal Nutrient Loading Interfere with the Long-Term Stability of Lake Restoration and Induce Sudden Re-eutrophication
Source: Environ Sci Technol. 2023 Feb 20;57(9):4003–13. doi: 10.1021/acs.est.2c07181 (PMC9997485; doi:10.1021/acs.est.2c07181)
Supplement: Supplementary file 1 — es2c07181_si_001.pdf [file es2c07181_si_001.pdf]

Supporting Information

Synergistic effects of warming and internal nutrient loading interfere with the long-term stability of lake restoration and induce sudden re-eutrophication

Xiangzhen Kong[1,2,*], Maria Determann[2], Tobias Kuhlmann Andersen[3], Carolina Cerqueira Barbosa[4], Tallent Dadi[2], Annette B.G. Janssen[5], Ma. Cristina Paule-Mercado[6], Diego Guimarães Florencio Pujoni[7], Martin Schultze[2], Karsten Rinke[2]

[1] State Key Laboratory of Lake Science and Environment, Nanjing Institute of Geography and Limnology, Chinese Academy of Sciences, 210008 Nanjing, China

[2] Department of Lake Research, Helmholtz Centre for Environmental Research - UFZ, 39114 Magdeburg, Germany

[3] Department of Ecoscience, Aarhus University, 8000 Aarhus, Denmark

[4] Zoology and Physiology Department, University of Wyoming, Laramie, WY 82071, USA

[5] Water Systems and Global Change Group, Wageningen University & Research, Droevendaalsesteeg 3, 6708 PB, Wageningen, the Netherlands

[6] Czech Academy of Sciences, Biology Centre, Institute of Hydrobiology, Na Sádkách 7, České Budějovice 37005, Czech Republic

[7] Laboratório de Limnologia, Ecotoxicologia e Ecologia Aquática, Instituto de Ciências Biológicas, Universidade Federal de Minas Gerais, Avenida Antônio Carlos 6627, Cep 31270-901, Belo Horizonte, MG, Brazil

[*]Corresponding author: Xiangzhen Kong (xzkong@niglas.ac.cn)

This supporting information document has 28 papers, including the text for additional details of the modeling approach with four subsections, six Tables (S1-S6), and ten Figures (S1-S10).

Table of Content

- **SI Text: Additional details of the modeling approach**

  S1: The "restart" and "manipulate" functions

  S2: Nutrient release from sediment

  S3: Export coefficient model

  S4: Model autocalibration with PARSAC

- **SI Table S1-S6:**

  S1: Model coefficients (slope *k* and intercept *b* using linear regression model) for the bias correction of the meteorological data among the weather stations on the buoy, DWD and ERA5.

  S2: Annual TN loading estimation for the Lake Barleber

  S3. Working steps for GOTM-WET calibration

  S4: Parameter calibrated values of GOTM-WET model

  S5: Threshold values of the correlation coefficient (*R*) and relative error (*RE*) for lake ecosystem model performance

  S6: Model evaluation (GOTM-WET) for Lake Barleber from 2018 to 2021

- **SI Figures S1-S10**

  S1: Meteorology data from 2015 to 2021

  S2: Meteorology data from 1979 to 2021

  S3: Meteorology projections used for the long-term model simulation from 1980 to 2018, collected from ISIMIP project under the 'piControl' (without anthropogenic climate change) and historical (factual)

  S4: Observed and simulated water temperature profiles from 2018 to 2021

  S5: Observed and simulated water quality data in the hypolimnion from 2018 to 2021

  S6: Observed and simulated water quality data in the epilimnion from 2018 to 2021

  S7: Observed and simulated biomass of daphnia and meso-zooplankton in the water column

  S8: Simulated water temperature and DO concentration in the water column from 1986 to 2021

  S9: Simulated TN, NO_3^--N and NH_4^--N concentration in the water column from 1986 to 2021

  S10: Scenario analyses from 2015 to 2017 before and after the re-eutrophication of Lake Barleber in 2016

SI Text

Additional details of the modeling approach

1. The "restart" and "manipulate" functions

To model the process of P precipitation, the P pool in the water column needs to be transported to the sediment, and the internal P flux determined by the diffusion rate needs to be reduced simultaneously. These are realized by using both the 'manipulation' and 'restart' functions of the GOTM-WET model (illustrated in the figure below).

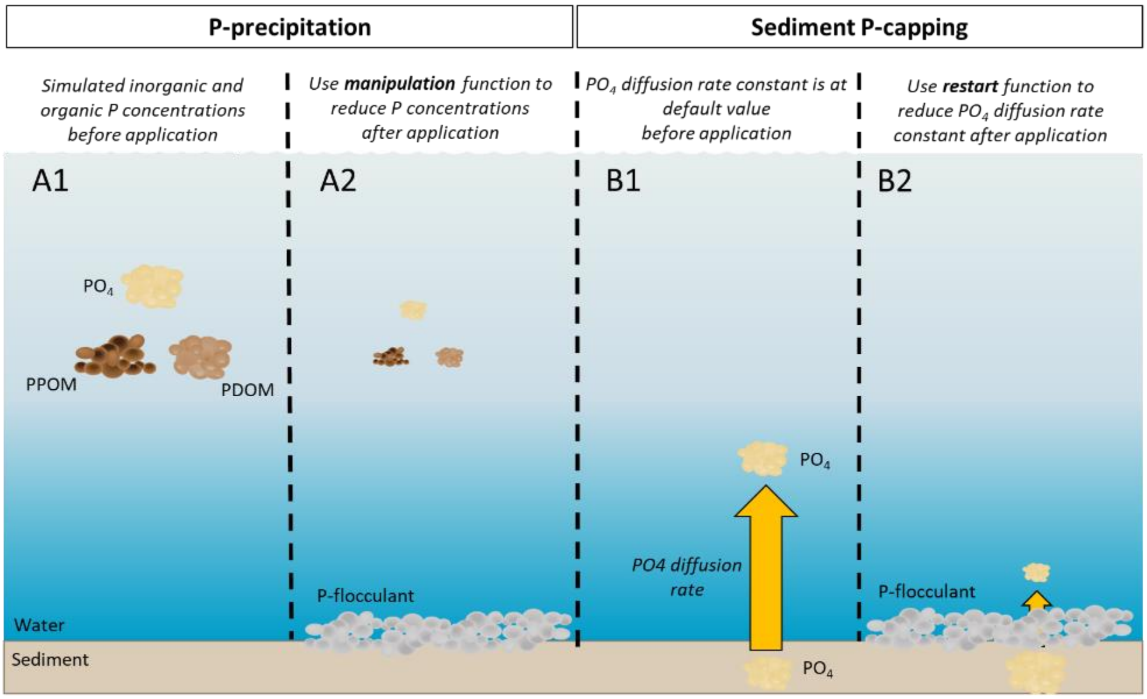

A conceptual figure of how this study modelled the effects of P precipitation application: A) P-precipitation and B) Sediment P-capping.

First, the 'manipulate' function allows that model to change in the state variables within simulation at certain time point (e.g. when the P precipitation was implemented). We force the P concentrations in the water column to be reduced to the observed level after the P precipitation, which is in concert with the observed abruptly reduced P concentration in the lake. Here, the manipulation refers to three P species in the water column modeled by WET,

i.e. dissolved reactive phosphate (*abiotic_water_sPO4W*), particulate organic P (*abiotic_water_sPPOMW*), and dissolved organic P (*abiotic_water_sPDOMW*). After P precipitation in both 1986 and 2019, all three P were reduced to a level below detection limits (sPO4W and sPDOMW: 0.003 $mg \cdot L^{-1}$; sPPOMW: 0.006 $mg \cdot L^{-1}$), which was reflected in the model by a namelist file named ‘manipulation.nml’ in the same working directory of the model (see Box 1 below as an example).

Second, the “restart” function allows the model to start running with the values for all state variables stored from a previous model run (i.e. similar to a ‘warm’ start). The function is enabled by setting the “restart -- load” domain as ‘true’ in the gotm.yaml configuration file. In this case, the model would initialize the simulation with state stored in the NetCDF file named “restart.nc”, which appears in the same working directory of the model configuration file. The “restart” function is utilized here to alter the parameter values related to internal P loading within one certain long-term simulation across ~40 years. The simulation was separated into several stages according to the implementation of P precipitation, and in each interval between stages, the ‘restart’ is used so that the model would continue simulating using the state by the end of previous stage, but with the new parameters, i.e. the reduced diffusion rate of P from the sediment, to mimic the effect of increasing P adsorbent agent in the lake sediment.

Box 1: Example of the namelist file ‘manipulation.nml’

```
&manipulation
   start = '1986-11-13 00:00:00',
   name = 'abiotic_water_sPO4W',
   action_type = 1,
   action = 0.003
   verbose = .true.
/
&manipulation
   start = '1986-11-13 00:00:00',
   name = 'abiotic_water_sPPOMW',
   action_type = 1,
   action = 0.006
   verbose = .true.
```

```
/
&manipulation
   start = '1986-11-13 00:00:00',
   name = 'abiotic_water_sPDOMW',
   action_type = 1,
   action = 0.003
   verbose = .true.
/
&manipulation
   start = '2019-08-15 00:00:00',
   name = 'abiotic_water_sPO4W',
   action_type = 1,
   action = 0.003
   verbose = .true.
/
&manipulation
   start = '2019-08-15 00:00:00',
   name = 'abiotic_water_sPPOMW',
   action_type = 1,
   action = 0.006
   verbose = .true.
/
&manipulation
   start = '2019-08-15 00:00:00',
   name = 'abiotic_water_sPDOMW',
   action_type = 1,
   action = 0.003
   verbose = .true.
/
```

2. Nutrient release from sediment

In GOTM-WET, dissolved inorganic phosphorus is transported from the sediment pore water to the upper water column via diffusion across the sediment-water interface, following the original PCLake model (Janse, 2005; pp 300). In most cases, phosphorus diffuses upward from sediment to water, i.e. nutrient release from the sediment that constitute the internal P loading of the lake ecosystems.

The equation for the P diffusion is:

$$tPDifPO4 = kPDifPO4 \cdot uFunTmDif \cdot cTurbDifNut \cdot bPorCorS \cdot \frac{(oPO4S - sPO4W)}{aDepthDif}$$

in which

$tPDifPO4$: diffusion flux of dissolved P from sediment to water [g P·m^{-2}·h^{-1}]

$kPDifPO4$: diffusion rate constant of dissolved P [m^{2}·h^{-1}] (default value: 2.6·10^{-6})

$uFunTmDif$: temperature correction of the diffusion [-], which is modeled using an *Arrhenius* temperature modifier: $1.02^{(uTm-20)}$, where *uTm* is the actual water temperature (℃)

$cTurbDifNut$: bioturbation factor of diffusion [-]

$bPorCorS$: sediment porosity corrected for tortuosity [-] (= $bPorS^{(bPorS + 1)}$, where *bPorS* is the porosity of sediment)

$oPO4S$ and $sPO4W$: P concentration in the sediment interstitial water and the water column of the lake, respectively

$aDepthDif$: diffusion distance [m], which is half of the active sediment depth (*cDepthS*, 0.1 m)

The effect of dissolved oxygen on P release is represented by the factor of $afOxySed$, which is calculated as:

$aDepthDif = aDepthOxySed / cDepthS$

in which

$aDepthOxySed$ is the oxygen penetration depth (m) described as a function of the oxygen concentration in the water, the oxygen diffusion rate and the sediment oxygen demand per volume of sediment.

$aDepthDif$ determines the P adsorption isotherm onto the inorganic matter in sediment, which affects the equilibrium of sediment inorganic P concentration in the interstitial water and particulates, thereby affecting the $oPO4S$. The model simulates the effects of oxygen on the P release such that when the sediment is aerobic, $oPO4S$ is reduced and so does the P release flux. Otherwise, when the sediment is anerobic, the equilibrium moves towards interstitial water and $oPO4S$ increases, so as the P release via diffusion.

For more detailed information regarding the sediment P release in the WET model, we refer to the technical document of the PCLake model (Janse, 2005), from which WET is developed.

The source code for WET is available at https://gitlab.com/WET/wet.

3. Export coefficient model

The export coefficient model is widely used to predict the loss of total nitrogen (TN) and phosphorus (TP) from a complex catchment to its drainage system (Johnes, 1996). The nutrient loading is calculated annually, as the sum of the individual loads from each source in the catchment. The model was originally developed in North America, and then adopted by the Organization for Economic Co-operation and Development (OECD) (Beaulac and Reckhow, 1982; OECD, 1982). The model considers various nutrient export sources, which is estimated as the product of source inputs and export coefficients as described below:

$$L = \sum_{i=1}^{n} E_i \cdot [A_i I_i] + p$$

where *L* is loss of nutrients; *E* is export coefficient for nutrient source *i*; *A* is area of catchment occupied by land use type *i*; *I* is input of nutrients to source *i*; *p* is input of nutrients from atmospheric deposition (e.g. precipitation).

For the catchment of Lake Barleber, TP loading has been determined as 600 kg $P \cdot a^{-1}$ from sediment release (during 2017 after re-eutrophication) and 78 kg $P \cdot a^{-1}$ from other sources including atmospheric deposition, groundwater input, and diffusive input (recreation activities e.g. bathing).

The export coefficient model is used to calculate TN loading. The sources include humans at the lake's surrounding area, and the wider catchment dominated by the cultivation of forest, grains and root vegetables. The TN loading estimation to the lake from these sources are described in Table S1 with more details.

Besides, dissolved silica loading is estimated based on the application of export coefficients model in central Europe (with a coefficient of 24.55 $kg \cdot ha^{-1} \cdot a^{-1}$ (Onderka et al., 2012).

4. Model autocalibration with PARSAC

The calibration of GOTM-WET model was implemented using the automatic calibration program PARSAC (https://bolding-bruggeman.com/portfolio/parsac/). This method has been successfully applied in several previous studies with GOTM-WET (Andersen et al., 2020; Chen et al., 2020; Kong et al., 2022). PARSAC uses the parallel direct search method termed Differential Evolution (Storn and Price, 1997). This method allows the model to estimate the global optimal of parameter values within the predefined ranges, via the optimization of a Maximum Likelihood multi-objective function calculated by both model outputs and field observations. Thus, such method is more advanced than the conventional manual trial-and-error calibration (Andersen et al., 2020).

In this study, the autocalibration procedure is conducted in a ‘bottom-up’ way with 6 steps (Table S3), in which different state variables and processes are selected with a working flow from lake physics (hydrodynamics), chemical (DO, nutrients) and ecological (plankton, mainly phytoplankton). The selection of parameters for the autocalibration was based on the model sensitivity analysis for WET model (Andersen et al., 2021), and the original PCLake model (Janse et al., 2010; Nielsen et al., 2014) and FABM-PCLake (Hu et al., 2016), where the WET is developed from. The total number of selected parameters for autocalibration is 161, which is provided in the supplementary Table S4.

In the beginning of the autocalibration, each selected parameter was assigned by a prior range derived from theoretical investigations (Andersen et al., 2020; Schnedler-Meyer et al., 2022). In each step, PARSAC runs for thousands of times towards a better model performance determined by a likelihood function (https://github.com/BoldingBruggeman/parsac), along with a posterior, narrower range of the parameters. The autocalibration stops in each step when the model performance could no longer be appreciably improved. Then, the posterior parameters would be propagated to the next step.

SI Tables

Table S1. Annual TN loading estimation for the Lake Barleber.

**Sources**	**Area/Number**	**Export coefficients of TN**	**Total amounts**	**References**
Humans	12,500 $ca \cdot a^{-1}$	0.36 $kg \cdot ca^{-1} \cdot a^{-1}$	4,500 $kg \cdot a^{-1}$	(Hannappel and Strom, 2020; Johnes, 1996)
Forest	289 ha	13.0 $kg \cdot ha^{-1} \cdot a^{-1}$	3,757 $kg \cdot a^{-1}$	(Johnes, 1996)
Grains	540 ha	19.1 $kg \cdot ha^{-1} \cdot a^{-1}$	10,314$kg \cdot a^{-1}$	(Johnes, 1996)
Root vegetation	308 ha	14.3 $kg \cdot ha^{-1} \cdot a^{-1}$	4,404.4 $kg \cdot a^{-1}$	(Johnes, 1996)
Atmospheric deposition	103 ha	18.1 $kg \cdot ha^{-1} \cdot a^{-1}$	1,864.3 $kg \cdot a^{-1}$	(Schröder et al., 2011)
Total	1,240ha	-	24,839.7 $kg \cdot a^{-1}$	-

Table S2. Model coefficients (slope *k* and intercept *b* using linear regression model) for the bias correction of the meteorological data among the weather station on the buoy, DWD and ERA5. The ERA5 data were first corrected against the DWD data available from 1979 to 2021. Then, for a subset of meteorological variables from the weather station data on the lake buoy available from 2018 to 2021, the DWD data were corrected against the weather station data and further applied to the ERA5 data. The corrected ERA5 data would be used for the model simulation.

**Variable (unit)**	**Explanation**	**Correct ERA5 against DWD** $Y_{DWD}=k \cdot X_{ERA5} + b$		**Correct DWD against Buoy** $Y_{buoy}=k \cdot X_{DWD} + b$	
		k	***b***	***k***	***b***
**u10 ($m \cdot s^{-1}$)**	wind speed at E-W direction	0.77	0.02	1.42	0.18
**v10 ($m \cdot s^{-1}$)**	wind speed at N-S direction	0.80	-0.10	1.25	0.07
**airp (hPa)**	air pressure	1.00	3.24	1.01	-8.13
**airt (°C)**	air temperature	1.00	0.02	0.96	0.45
**dp (°C)**	dew-point temperature	1.00	0.02	-	-
**relH (-)**	relative humidity	-	-	0.85	9.87
**cloud (-)**	cloud cover	0.71	0.18	-	-
**swr ($W \cdot m^{-2}$)**	solar radiation	0.95	14.29	1.00	2.38
**pp ($mm \cdot h^{-1}$)**	precipitation	0.84	0.01	0.85	0.04

Table S3. Work steps for GOTM-WET calibration in Lake Barleber including 6 steps for different model state variables linked to various processes. Note that the latter steps include calibrated parameters from the previous steps but with narrowed ranges. The table is adapted from Andersen et al. (2020).

Step	Processes	State variables
1	lake hydrodynamic processes	water temperature
2	mineralization	dissolved oxygen (DO)
3	denitrification and nitrification	nitrogen (NO_3^--N, NH_4^--N, and TN)
4	P interactions in water column and sediment	phosphorus (PO_4^{3-}-P and TP)
5	phytoplankton seasonal trends	Chl a concentration, phytoplankton composition and dry mass
6	all nutrient and plankton dynamics	Step 2-5

Table S4. Parameter explanation and the calibrated values of GOTM-WET model from this study compared with the values from literature.

(see separated Excel file named '**si_Table_S4.xlsx**')

Table S5. Threshold values of the correlation coefficient (*R*) and relative error (*RE*) for lake ecosystem model performance for various variables. The threshold values are based on the statistical summary from Arhonditsis and Brett (2004), and the four categories of the performance are defined as the percentiles values among the modeling results in literature: Fair (<20%), Satisfactory (20-50%), Good (50-80%), Excellent (80-100%).

Variables (Unit)	R				RE			
	Fair	Satisfactory	Good	Excellent	Fair	Satisfactory	Good	Excellent
**Water Temp. (℃)**	0.65	0.79	0.96	0.98	15%	11%	7%	4%
**TN ($mg·L^{-1}$)**	0.22	0.36	0.62	0.88	101%	77%	48%	30%
**NO_3-N ($mg·L^{-1}$)**	0.32	0.61	0.82	0.95	88%	68%	36%	18%
**NH_4-N ($mg·L^{-1}$)**	0.22	0.36	0.62	0.88	101%	77%	48%	30%
**TP ($mg\ L^{-1}$)**	0.26	0.36	0.69	0.89	84%	69%	42%	26%
**SRP ($mg·L^{-1}$)**	0.26	0.36	0.69	0.89	84%	69%	42%	26%
**Particulate P ($mg·L^{-1}$)**	0.26	0.36	0.69	0.89	84%	69%	42%	26%
**DO ($mg·L^{-1}$)**	0.58	0.72	0.84	0.94	22%	11%	7%	4%
**Silica ($mg·L^{-1}$)**	0.45	0.59	0.78	0.89	64%	55%	37%	30%
**Chl-a ($mg·m^{-3}$)**	0.28	0.40	0.69	0.87	79%	66%	44%	26%
**Cyanobact. Chl-a ($mg·m^{-3}$)**	0.28	0.40	0.69	0.87	79%	66%	44%	26%
**Diatom Chl-a ($mg·m^{-3}$)**	0.28	0.40	0.69	0.87	79%	66%	44%	26%

Arhonditsis, G. B.; Brett, M. T., Evaluation of the current state of mechanistic aquatic biogeochemical modeling. *Mar. Ecol. Prog. Ser.* **2004,** *271*, 13-26.

Table S6. Model evaluation (GOTM-WET) for Lake Barleber from 2018 to 2021 based on volume-weighted data in the whole water column. Evaluation criteria include correlation coefficient (*R*) and relative error (*RE*) of observed and simulated data in both calibration and validation. The colors represent various level of model performance, and the threshold are provided in Table S5. The final remarks are defined as the intermediate results of *R* and *RE*, for example, a 'satisfactory' *R* and 'excellent' *RE* results in a 'Good' final remark.

Fair	Satisfactory	Good	Excellent

Variables (Unit)	***R***		***RE***		***Final remarks***	
	Calib.	***Valid.***	***Calib.***	***Valid.***	***Calib.***	***Valid.***
Water Temp. (°C)	0.97	0.98	5.9	-0.2	Good	Excellent
TN (mg·L^{-1})	0.41	0.62	13.3	-25.7	Good	Good
NO_3-N (mg·L^{-1})	0.44	0.48	36.2	43.6	Fair	Fair
NH_4-N (mg·L^{-1})	0.52	0.67	17.3	2.0	Good	Good
TP (mg·L^{-1})	0.76	0.89	12.4	24.7	Good	Excellent
SRP (mg·L^{-1})	0.78	0.84	16.4	3.9	Good	Good
PP (mg L^{-1})	0.53	0.65	-20.1	13.7	Good	Good
DO (mg·L^{-1})	0.79	0.78	-1.7	0.8	Good	Good
Silica (mg·L^{-1})	0.82	0.69	-29.3	6.1	Good	Good
Chl-a (mg·m^{-3})	0.47	0.41	19.7	18.3	Good	Good
Cyanobacteria Chl-a (mg·m^{-3})	0.55	0.42	4.6	3.0	Good	Good
Diatom Chl-a (mg·m^{-3})	0.48	0.41	28.4	19.90	Satisfactory	Good

Note:

Calib.: Model calibration using data from 2018.6 to 2019.6 before the P precipitation;

Valid.: Model validation using data from 2019.7 to 2021.9 after the P precipitation.

SI Figures

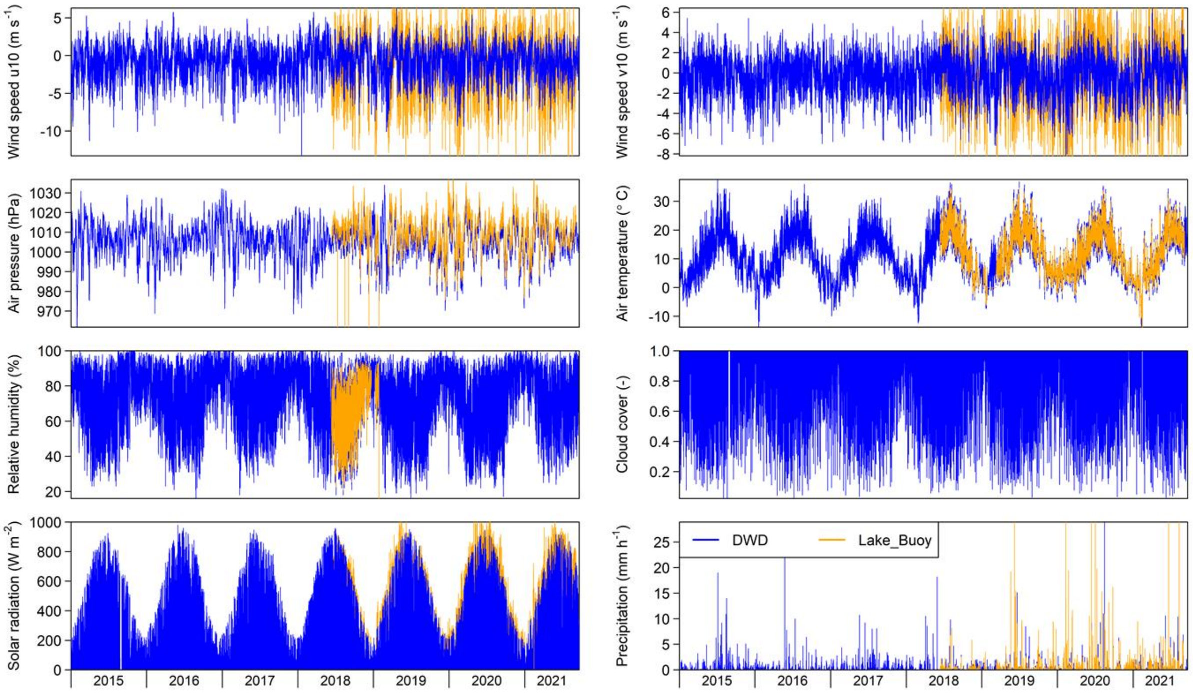

Figure S1. Meteorology data from 2015 to 2021 at hourly basis from the German weather service (DWD) "Magdeburg" station (~15km from the lake), compared with the buoy weather station data collected from June 2018 to December 2021. Variables include wind speed (at both u and v direction), air pressure, air temperature, relative humidity, cloud cover, solar radiation and precipitation.

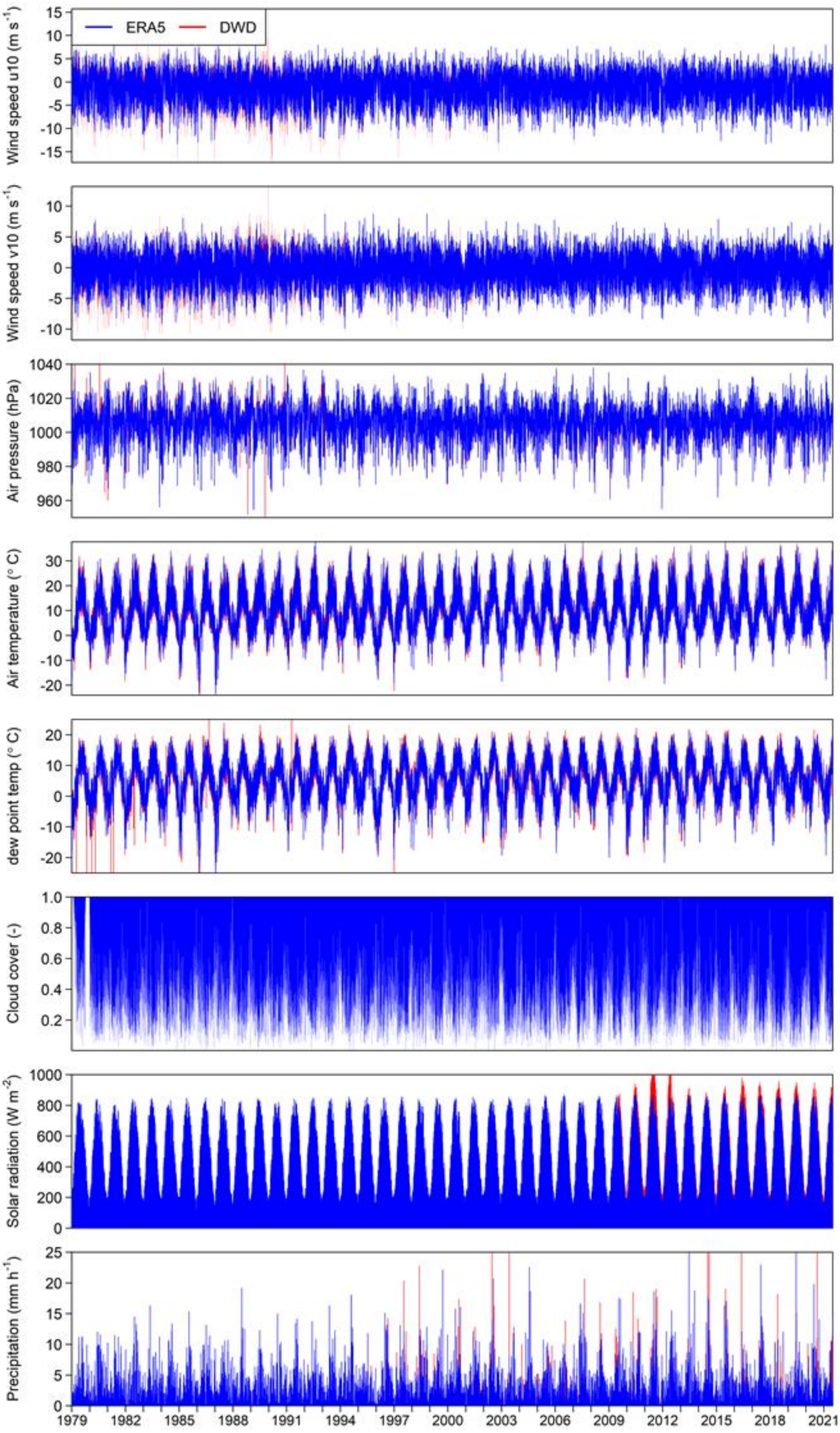
ERA5
DWD
Wind speed u10 (m s-1)
Wind speed v10 (m s-1)
Air pressure (hPa)
Air temperature (° C)
dew point temp (° C)
Cloud cover (-)
Solar radiation (W m-2)
Precipitation (mm h-1)
1979
1982
1985
1988
1991
1994
1997
2000
2003
2006
2009
2012
2015
2018
2021

Figure S2. Meteorology data from 1979 to 2021 at hourly basis from the German weather service (DWD) "Magdeburg" station (~15km from the lake), compared with the ERA5 data collected in the same time duration. Variables include wind speed (at both u and v direction), air pressure, air temperature, relative humidity, cloud cover, solar radiation and precipitation.

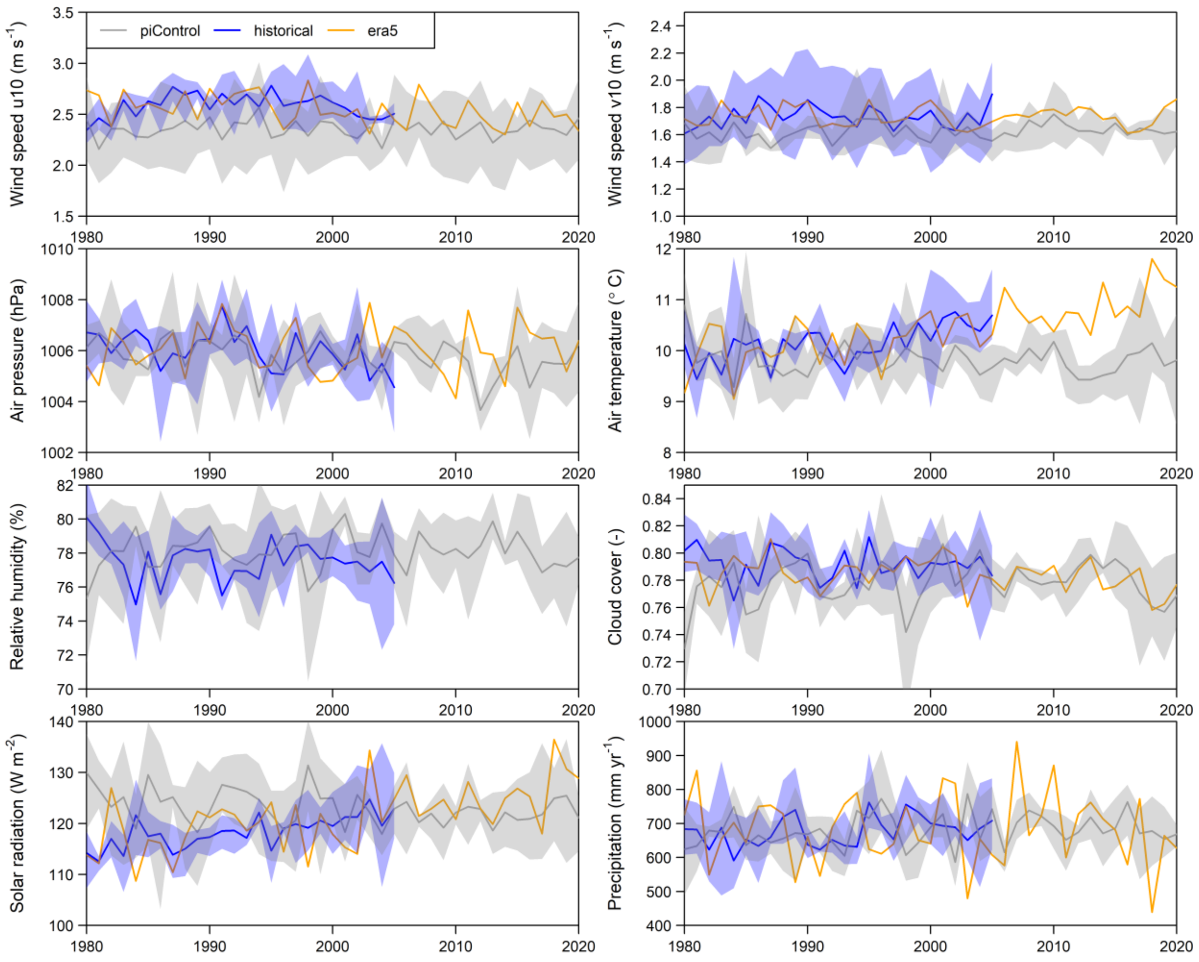

Figure S3. Meteorology projections used for the long-term model simulation from 1980 to 2018, collected from ISIMIP project under the ‘piControl’ (without anthropogenic climate change), historical (factual). The dataset was at hourly basis and aggregated to annual basis to compare to the ERA5 data (2017-2021). The ISIMIP data were derived using three Global Climate Models (GCMs) (HadGEM2-ES, IPSL-CM5A-LR, and GFDL-ESM2M). The solid lines are the average value, and the shaded areas are the variation calculated from the projections of the three GCMs.

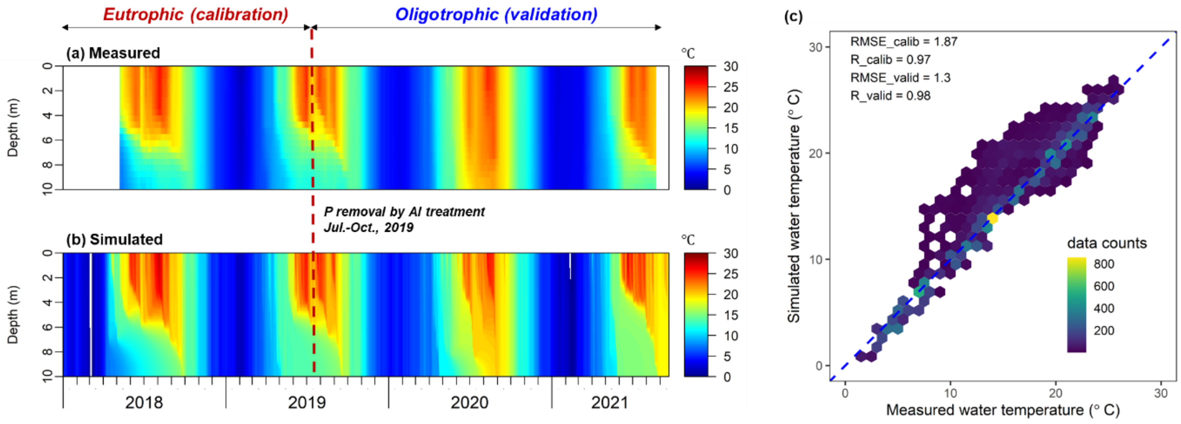

Figure S4. (a and b) Observed and simulated water temperature profiles from 2018 to 2021 (Sept. 20) in the Lake Barleber. Data from June 2018 to June 2019 are used for model calibration, and data afterwards are used for model validation. (c) Measured against simulated water temperature data shown as data counts density, and the *RMSE* and *R* values for both calibration and validation periods.

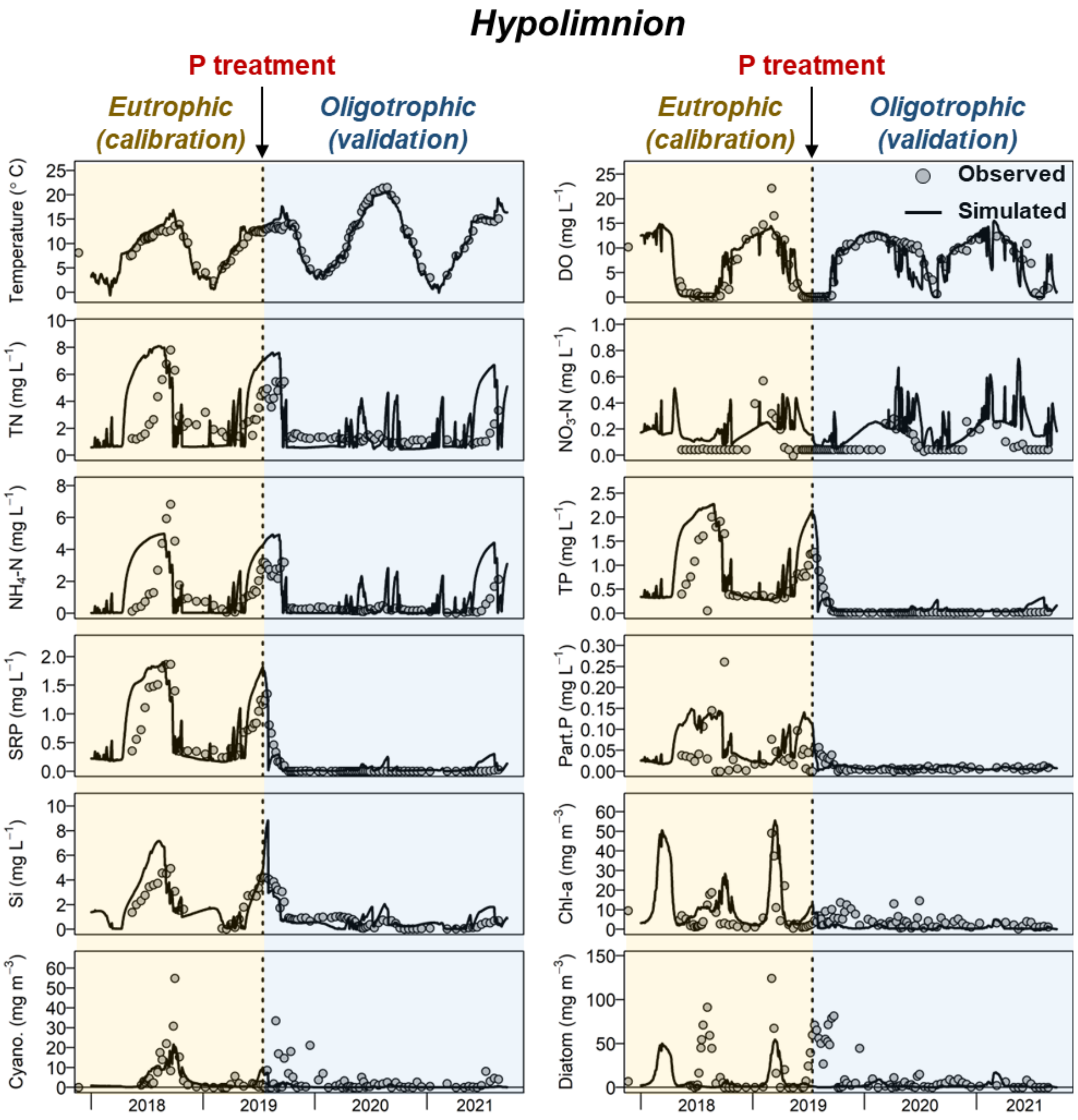

Figure S5. Volume-weighted values of observed and simulated water quality data in the hypolimnion from 2018 to 2021 in Lake Barleber. The model was calibrated against the data between June 2018 and June 2019 when the lake was eutrophic (before the P precipitation), and validated against the data between July 2019 and November 2021 when the lake was oligotrophic.

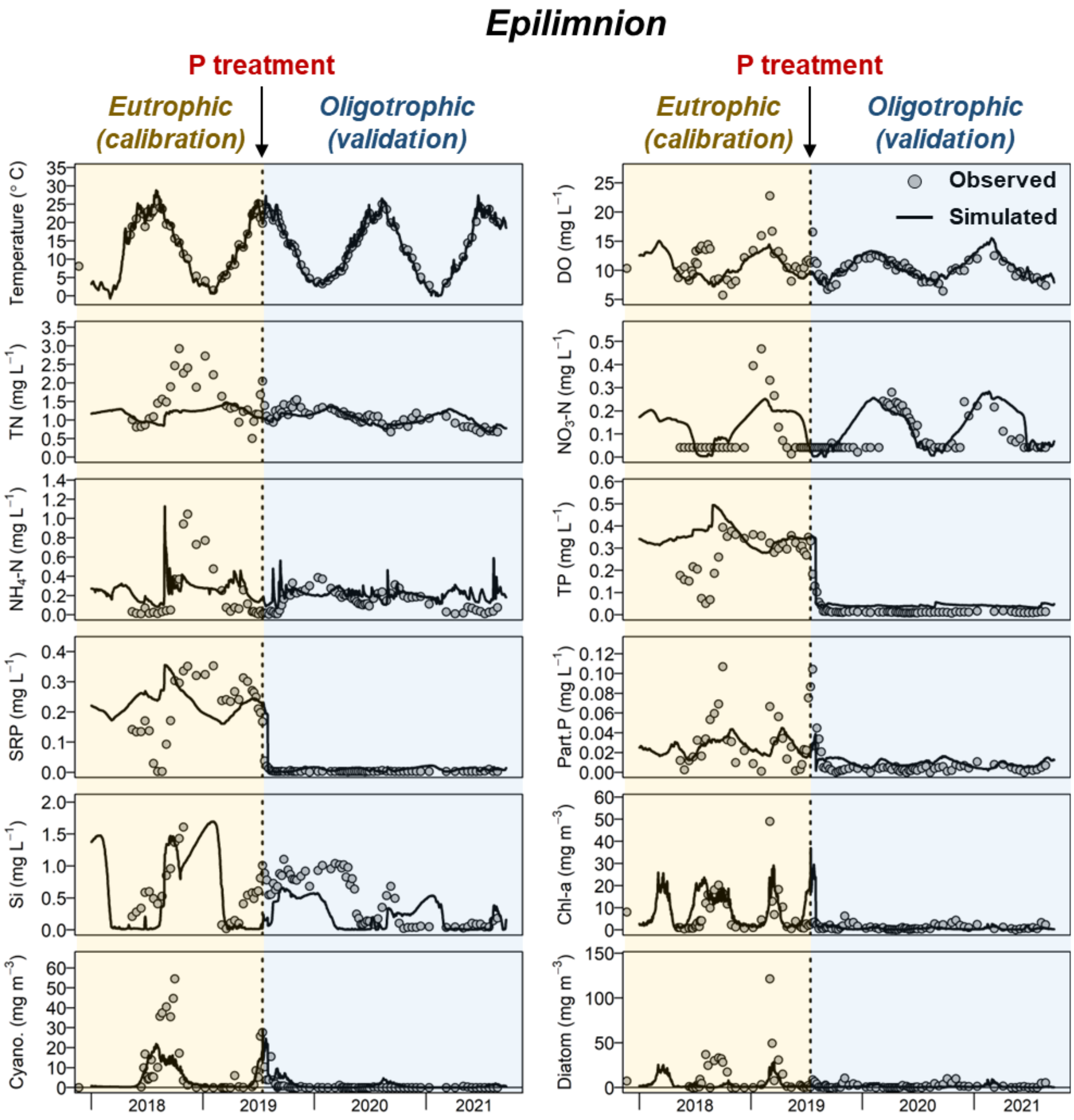

Figure S6. Volume-weighted values of observed and simulated water quality data in the epilimnion from 2018 to 2021 in Lake Barleber. The model was calibrated against the data between June 2018 and June 2019 when the lake was eutrophic (before the P precipitation), and validated against the data between July 2019 and November 2021 when the lake was oligotrophic.

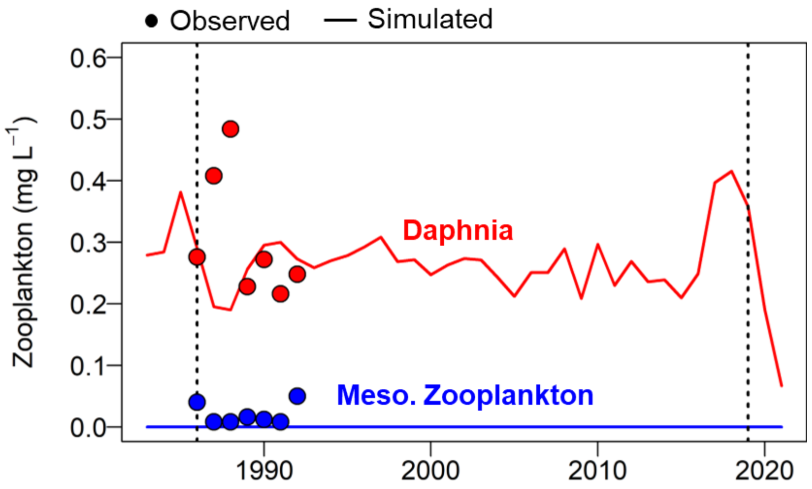

Figure S7. Comparison between observed (1986-1992) and simulated (1986-2021) biomass of daphnia and meso-zooplankton in the water column in Lake Barleber. Volume-weighted values of observed and simulated data across the water column are provided. Data collected from Rönicke et al. (2021).

Rönicke, H., Frassl, M.A., Rinke, K., Tittel, J., Beyer, M., Kormann, B., Gohr, F. and Schultze, M. 2021. Suppression of bloom-forming colonial cyanobacteria by phosphate precipitation: A 30 years case study in Lake Barleber (Germany). Ecol. Eng. 162, 106171.

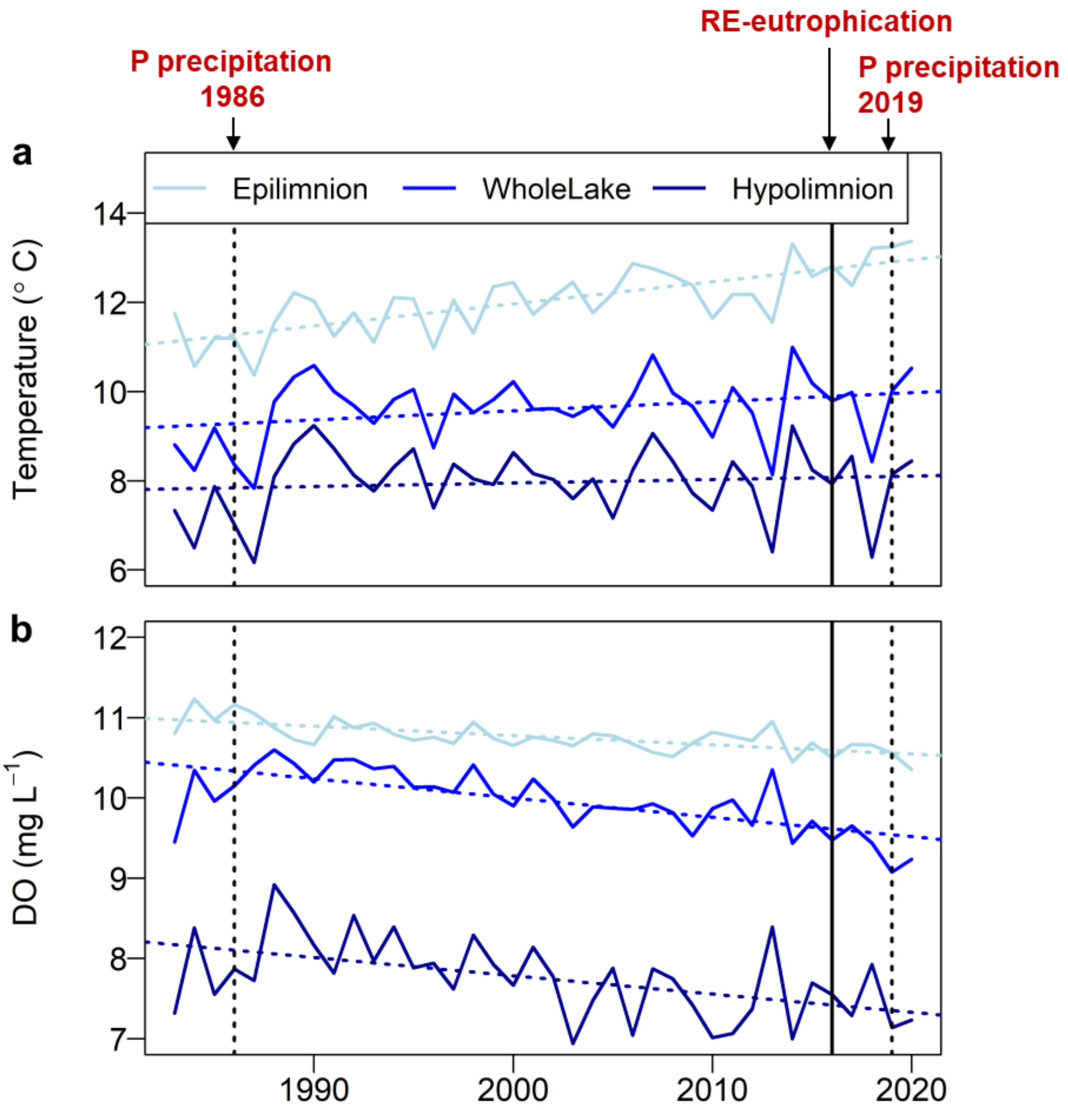

Figure S8. Simulated (a) water temperature and (b) DO concentration in the water column from 1986 to 2021 (annual average) in Lake Barleber. Volume-weighted values of epilimnion, hypolimnion and the whole-lake-averaged are provided. The dashed horizontal lines are the linear regression of the simulated data.

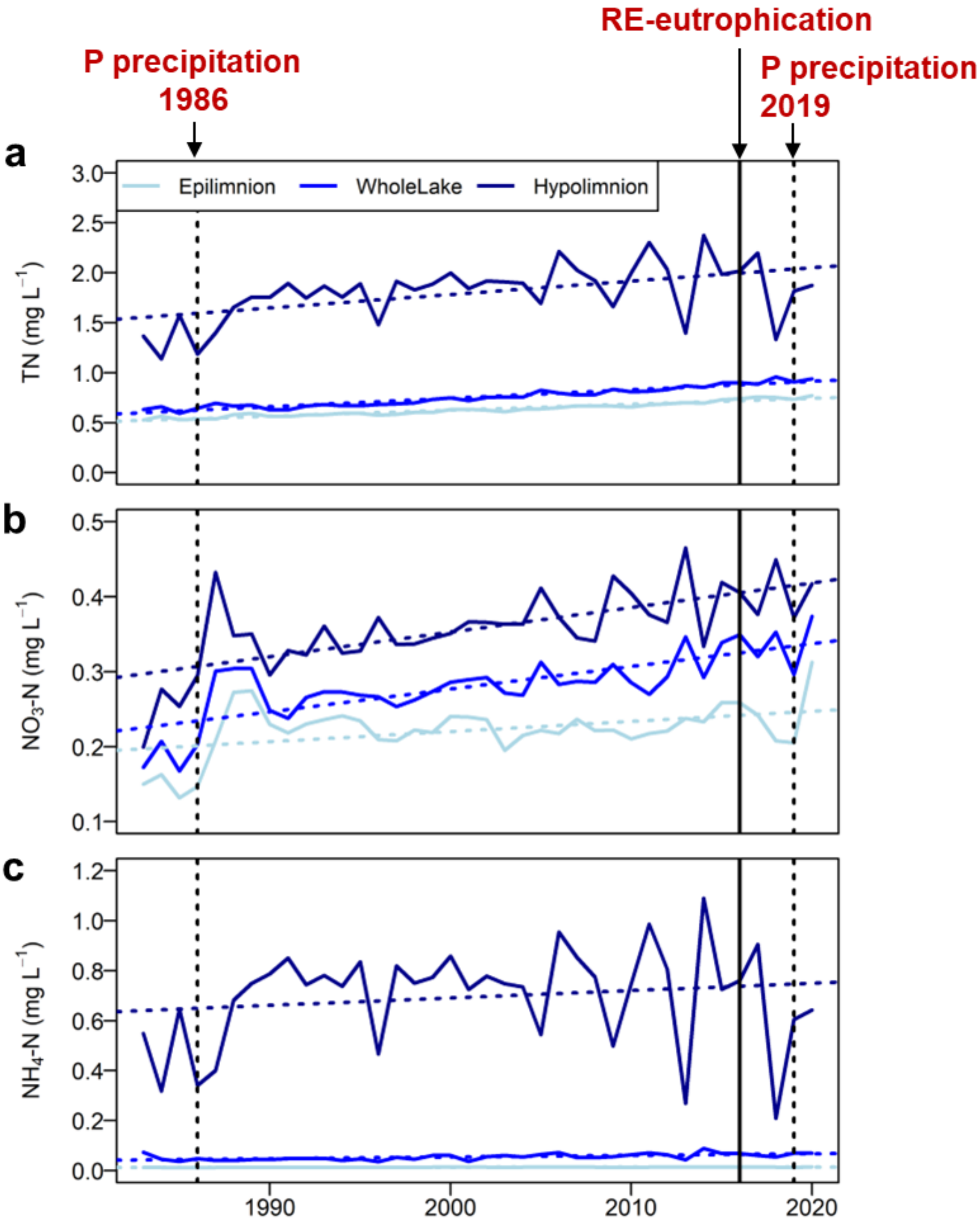

Figure S9. Simulated (a) TN, (b) NO3-N and (c) NH4-N concentration in the water column from 1986 to 2021 (annual average) in Lake Barleber. Volume-weighted values of epilimnion, hypolimnion and the whole-lake-averaged are provided. The dashed horizontal lines are the linear regression of the simulated data.

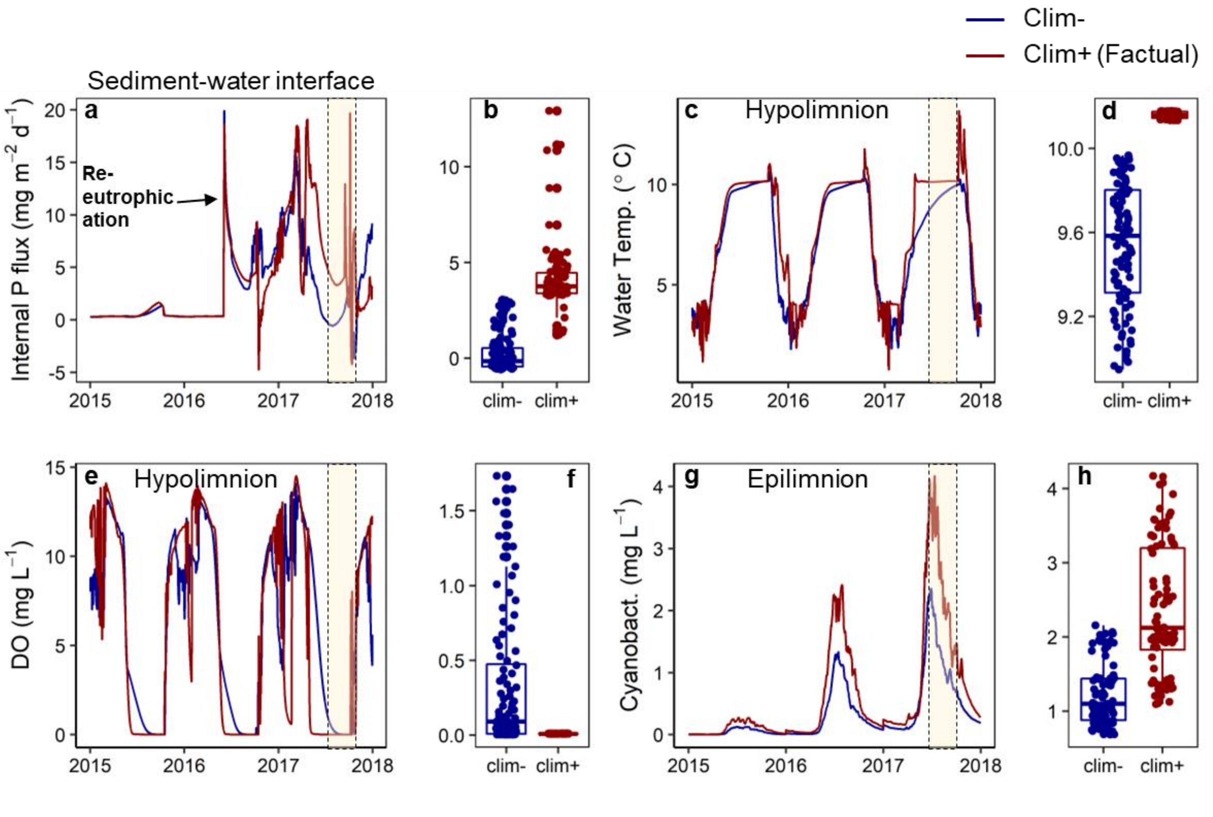

Figure S10. Scenario analyses from 2015 to 2017 before and after the 2016 re-eutrophication of Lake Barleber. (a and b) Internal phosphorus release flux averaged over the water-sediment interface. (c and d) Water temperature at the hypolimnion. (e and f) Dissolved oxygen concentration at the hypolimnion. (g and h) Cyanobacterial biomass at the epilimnion. For the time series plots (a, c, e and g), model simulated results are shown at a daily interval for climate scenarios with ("clim+") and without ("clim-") anthropogenic climate change (both under "intP+" condition). "Factual" represents the scenario of actual climate condition (with climate change). For the boxplot panels (b, d, f, and h), the data correspond to the period of July to September in 2017 as a representative of re-eutrophication, which is highlighted by a yellow strip in the time series panels.

References

Andersen, T.K., Bolding, K., Nielsen, A., Bruggeman, J., Jeppesen, E. and Trolle, D. 2021. How morphology shapes the parameter sensitivity of lake ecosystem models. Environ. Model. Software 136, 104945.

Andersen, T.K., Nielsen, A., Jeppesen, E., Hu, F., Bolding, K., Liu, Z., Søndergaard, M., Johansson, L.S. and Trolle, D. 2020. Predicting ecosystem state changes in shallow lakes using an aquatic ecosystem model: Lake Hinge, Denmark, an example. Ecol. Appl. 30(7), e02160.

Beaulac, M.N. and Reckhow, K.H. 1982. An examination of land use: nutrient export relationships. Water Resources Bulletin 18(6), 1013-1024.

Chen, W., Nielsen, A., Andersen, T.K., Hu, F., Chou, Q., Søndergaard, M., Jeppesen, E. and Trolle, D. 2020. Modeling the ecological response of a temporarily summer-stratified lake to extreme heatwaves. Water 12(1), 94.

Hannappel, S. and Strom, A. 2020. Method to determine the amount of phosphorous entering Barleber See near Magdeburg via groundwater. Korrespondenz Wasserwirtschaft (in German) 13, 24-30.

Hu, F., Bolding, K., Bruggeman, J., Jeppesen, E., Flindt, M., van Gerven, L., Janse, J., Janssen, A., Kuiper, J. and Mooij, W. 2016. FABM-1 PCLake-linking aquatic ecology with hydrodynamics. Geosci. Model Dev. 9(2), 2271–2278.

Janse, J.H. (2005) Model studies on the eutrophication of shallow lakes and ditches [Doctoral dissertation]. Wageningen University, Wageningen, The Netherlands.

Janse, J.H., Scheffer, M., Lijklema, L., Van Liere, L., Sloot, J.S. and Mooij, W.M. 2010. Estimating the critical phosphorus loading of shallow lakes with the ecosystem model PCLake: Sensitivity, calibration and uncertainty. Ecol. Model. 221(4), 654-665.

Johnes, P.J. 1996. Evaluation and management of the impact of land use change on the nitrogen and phosphorus load delivered to surface waters: the export coefficient modelling approach. J. Hydrol. 183(3-4), 323-349.

Kong, X., Ghaffar, S., Determann, M., Friese, K., Jomaa, S., Mi, C., Shatwell, T., Rinke, K. and Rode, M. 2022. Reservoir water quality deterioration due to deforestation emphasizes the indirect effects of global change. Water Res., 118721.

Nielsen, A., Trolle, D., Bjerring, R., Søndergaard, M., Olesen, J.E., Janse, J.H., Mooij, W.M. and Jeppesen, E. 2014. Effects of climate and nutrient load on the water quality of shallow lakes assessed through ensemble runs by PCLake. Ecol. Appl. 24(8), 1926-1944.

OECD 1982. Eutrophication of Waters . Monitoring , Assessment and Control Final Report, OECD Cooperative Program on Monitoring of Inland Waters (Eutrophication Control) , Environment Directorate, OECD, Paris, 154.

Onderka, M., Wrede, S., Rodny, M., Pfister, L., Hoffmann, L. and Krein, A. 2012. Hydrogeologic and landscape controls of dissolved inorganic nitrogen (DIN) and dissolved silica (DSi) fluxes in heterogeneous catchments. J. Hydrol. 450, 36-47.

Rönicke, H., Frassl, M.A., Rinke, K., Tittel, J., Beyer, M., Kormann, B., Gohr, F. and Schultze, M. 2021. Suppression of bloom-forming colonial cyanobacteria by phosphate precipitation: A 30 years case study in Lake Barleber (Germany). Ecol. Eng. 162, 106171.

Schnedler-Meyer, N.A., Andersen, T.K., Hu, F.R.S., Bolding, K., Nielsen, A. and Trolle, D. 2022. Water Ecosystems Tool (WET) 1.0 – a new generation of flexible aquatic ecosystem model. Geosci. Model Dev. 2022(15), 3861–3878.

Schröder, W., Holy, M., Pesch, R., Harmens, H. and Fagerli, H. 2011. Mapping background values of atmospheric nitrogen total depositions in Germany based on EMEP deposition modelling and the European Moss Survey 2005. Environmental Sciences Europe 23(1), 1-9.

Storn, R. and Price, K. 1997. Differential evolution–a simple and efficient heuristic for global optimization over continuous spaces. J. Glob. Optim. 11(4), 341-359.
